# Assembly of higher-order SMN oligomers is essential for metazoan viability and requires an exposed structural motif present in the YG zipper dimer

**DOI:** 10.1093/nar/gkab508

**Published:** 2021-06-28

**Authors:** Kushol Gupta, Ying Wen, Nisha S Ninan, Amanda C Raimer, Robert Sharp, Ashlyn M Spring, Kathryn L Sarachan, Meghan C Johnson, Gregory D Van Duyne, A Gregory Matera

**Affiliations:** Department of Biochemistry & Biophysics, Perelman School of Medicine, University of Pennsylvania, Philadelphia, PA 19105-6059, USA; Integrative Program for Biological & Genome Sciences, Lineberger Comprehensive Cancer Center, University of North Carolina, Chapel Hill, NC 27599, USA; Department of Biochemistry & Biophysics, Perelman School of Medicine, University of Pennsylvania, Philadelphia, PA 19105-6059, USA; Integrative Program for Biological & Genome Sciences, Lineberger Comprehensive Cancer Center, University of North Carolina, Chapel Hill, NC 27599, USA; Curriculum in Genetics and Molecular Biology, Department of Genetics, University of North Carolina, Chapel Hill, NC 27599, USA; Department of Biochemistry & Biophysics, Perelman School of Medicine, University of Pennsylvania, Philadelphia, PA 19105-6059, USA; Integrative Program for Biological & Genome Sciences, Lineberger Comprehensive Cancer Center, University of North Carolina, Chapel Hill, NC 27599, USA; Department of Biology, University of North Carolina, Chapel Hill, NC 27599, USA; Department of Biochemistry & Biophysics, Perelman School of Medicine, University of Pennsylvania, Philadelphia, PA 19105-6059, USA; Department of Biology, University of North Carolina, Chapel Hill, NC 27599, USA; Department of Biochemistry & Biophysics, Perelman School of Medicine, University of Pennsylvania, Philadelphia, PA 19105-6059, USA; Integrative Program for Biological & Genome Sciences, Lineberger Comprehensive Cancer Center, University of North Carolina, Chapel Hill, NC 27599, USA; Curriculum in Genetics and Molecular Biology, Department of Genetics, University of North Carolina, Chapel Hill, NC 27599, USA; Department of Biology, University of North Carolina, Chapel Hill, NC 27599, USA

## Abstract

Protein oligomerization is one mechanism by which homogenous solutions can separate into distinct liquid phases, enabling assembly of membraneless organelles. Survival Motor Neuron (SMN) is the eponymous component of a large macromolecular complex that chaperones biogenesis of eukaryotic ribonucleoproteins and localizes to distinct membraneless organelles in both the nucleus and cytoplasm. SMN forms the oligomeric core of this complex, and missense mutations within its YG box domain are known to cause Spinal Muscular Atrophy (SMA). The SMN YG box utilizes a unique variant of the glycine zipper motif to form dimers, but the mechanism of higher-order oligomerization remains unknown. Here, we use a combination of molecular genetic, phylogenetic, biophysical, biochemical and computational approaches to show that formation of higher-order SMN oligomers depends on a set of YG box residues that are not involved in dimerization. Mutation of key residues within this new structural motif restricts assembly of SMN to dimers and causes locomotor dysfunction and viability defects in animal models.

## INTRODUCTION

Oligomeric proteins represent a significant fraction of cellular proteomes in all three domains of life. Self-interaction (homo-oligomerization) is a widespread and well-established feature of soluble proteins, occurring within a majority of known macromolecular assemblies (1). Protein oligomerization provides several functional benefits [reviewed in (2,3)], not least of which is the potential for forming novel and multivalent interaction surfaces that are not present in the monomer. Oligomerization can thus alter protein stability, enzymatic activity, and allosteric interactions; indeed, the manifold opportunities for adding new layers of regulation are too numerous to list.

The Survival Motor Neuron (SMN) protein forms the oligomeric core of a multiprotein complex that chaperones the biogenesis of small nuclear ribonucleoproteins (snRNPs) required for pre-mRNA splicing (4,5). Together with its Gemin protein partners (6), the SMN complex is also thought to participate in many other important cellular processes, including RNA transport, translation, endocytosis, cytoskeletal maintenance, and intracellular signaling (7–10). Notably, SMN is a key component of RNP-rich, phase-separated cellular domains known as stress granules (11) and Cajal bodies (12). Homozygous mutation or deletion of the human *SMN1* gene causes a devastating neuromuscular disease called Spinal Muscular Atrophy (SMA) (13,14). Most SMN orthologs have three conserved domains: an N-terminal region responsible for binding to Gemin2, a centrally located Tudor domain important for binding to Sm-class splicing factors, and a C-terminal YG box region that mediates self-interaction (15,16). There are two paralogous copies of *SMN* in humans, *SMN1* and *SMN2* (13). Non-human primate and other eukaryotic genomes have only a single-copy *Smn* gene (17), deletion of which is lethal in every species studied to date (18). Note that the Tudor domain of SMN is not essential for eukaryotic viability, as this domain is missing in certain phyla (e.g. fungi and trypanosoma). However, the YG box is present in all SMN orthologs identified to date and this domain is also important for the protein to associate with Gemin3 and Gemin8 (19–21).

The extent to which defects in SMN oligomerization contribute to the pathophysiology of SMA is not yet known. Roughly half of all SMA-causing missense mutations in *SMN1* are located within the YG box (22), and the predominant protein isoform expressed from the *SMN2* gene contains a truncation of this domain (23). Despite the widely held belief that oligomerization of SMN is important for its function, there is no direct evidence that higher-order (*n* > 2) multimers are actually necessary for SMN to carry out its activities. To better understand how the SMN protein forms oligomers, we carried out a detailed structure-function analysis of the YG box self-interaction domain. Using a broad spectrum of experimental approaches and model systems, we find that formation of higher-order SMN oligomers depends on specific YG box residues that are not involved in dimerization. These identified residues constitute a second structural motif that is not only required for SMN oligomerization *in vitro*, but also for organismal viability, longevity, and locomotor function *in vivo*.

## MATERIALS AND METHODS

### Protein expression purification and reconstitution of SMN•Gemin2

Human and nematode SMN•G2 complexes were purified as previously described (24,25). SMN•Gemin2 complex was produced by co-expression of SMN (residues 1–294) and Gemin2 (12-280) fused to a C-terminal Mxe intein (New England Biolabs (NEB), Beverly, MA, USA) containing a hexahistidine tag. Both coding regions were cloned into pETDuet (Novagen, Madison, WI, USA) and soluble SMN•Gemin2 complex was obtained following induction with 500 mM IPTG in BL21(DE3) cells for 16 h at 18°C. The complex was purified by Ni-NTA Superflow (Qiagen, Germantown MD) and chitin bead (NEB) chromatography at 4°C, following the vendor's protocols. SMN•Gemin2 complex was further purified on a Superdex 200 16/60 column at 20°C using 20 mM Tris-HCl pH7.5, 400 mM NaCl, 5 mM DTT, followed by spin column concentration (Millipore). SMNΔ5-Gemin2 complexes were produced using a similar protocol, except that SMNΔ5 was expressed from pCDFDuet and Gemin2-Mxe-His6 was produced from pETDuet together in BL21(DE3) and the final sizing column was performed using a Superdex 200 10/30 column at 4°C. Yeast SMN•G2 complexes were also purified as previously described (24).

The *Drosophila* SMN*•*Gemin2 complex was produced by separately expressing and purifying the two proteins and then reconstituting the complex *in vitro*. dmGemin2 was fused to a C-terminal Mxe intein (NEB) containing a hexahistidine tag, expressed in BL21(DE3) for 16 h at 18°C, and purified using Ni-NTA Superflow (Qiagen) and chitin bead (NEB) chromatography at 4°C following the vendor's protocols. Purified protein was further purified on a Superdex 200 16/60 column at 20°C using 20 mM Na/KPO4 pH 7.0, 300 mM NaCl, and 10 mM β-ME. Similarly, dmSMN was fused with an N-terminal His7-Flag-Sumo (HFS) tag and cloned into a pCDFDuet expression vector and expressed in BL21(DE3) for 2 h at 37°C following induction with 500 mM IPTG. Bacterial pellets were lysed in 100 mM NaKPO_4_, 10 mM Tris, 6M guanidine•HCL, 10 mM Imidazole, and 10 mM β-ME (final pH 8.0). After binding to Ni-NTA Superflow (Qiagen) resin, the column was subsequently washed with 15 c.v. of 100 mM Na/KPO_4_ (pH 7.0), 150 mM NaCl, 8M urea, 10 mM Imidazole, and 10 mM β-ME (final pH 7.7), followed by 20 c.v of 50 mM Na/KPO_4_ pH 7.0, 500 mM NaCl, 20 mM Imidazole, and 10 mM β-ME. Protein was eluted in 50 mM Na/KPO_4_ (pH 7.0), 400 mM NaCl, 300 mM Imidazole, and 10 mM β-ME.

A two molar excess of purified dmGemin2 was added and the N-terminal HFS tag was liberated from SMN by overnight cleavage with SUMO protease Ulp1 (Life Sensors) at 4°C. After passage through a second Ni-NTA column to capture fusion protein, the reconstituted dmSMN*•*G2 complex was purified on a on a Superdex 200 16/60 column at 20°C using 20 mM Na/KPO_4_ pH 7.0, 300 mM NaCl and 1 mM DTT, followed by spin column concentration (Millipore).

### Purification of MBP-dmSMN^186-220^

MBP-dmSMN^186-220^ was expressed pETDuet at 37°C and purified on amylose resins (New England Biolabs) followed by Superdex-200 sizing (G.E. Healthcare). Proteins were stored in 20 mM Na/KPO_4_ pH 7.0, 300 mM NaCl, 10 mM β-ME and 10% glycerol at –80°C.

### Size-exclusion chromatography in-line with multiangle light scattering (SEC-MALS)

Analyses were performed as previously described (26). Experiments were performed with a Superdex 200 10/300 GL column (GE Healthcare, Waukesha, WI, USA) at 0.5 ml/min at room temperature in 50 mM HEPES-NaOH pH 7.5, 250 mM NaCl and 5 mM DTT. The column was calibrated using the following proteins (Bio-Rad Laboratories, Hercules, CA, USA): thyroglobulin (670 kDa, *R*_S_ = 85 Å), γ-globulin (158 kDa, *R*_S_ = 52.2 Å), ovalbumin (44 kDa, *R*_S_ = 30.5 Å), myoglobin (17 kDa, *R*_S_ = 20.8 Å) and Vitamin B12 (1,350 Daltons). Blue-Dextran (Sigma-Aldrich, St. Louis, MO, USA) was used to define the void volume of the column.

Absolute molar mass of the proteins studied were determined using multi-angle light scattering coupled in-line with size-exclusion chromatography. Light scattering from the column eluant was recorded at 16 different angles using a DAWN-HELEOS MALS detector (Wyatt Technology Corporation, Santa Barbara, CA, USA) operating at 658 nm. The detectors at different angles were calibrated using the monomer peak of Fraction V bovine serum albumin (Sigma). Protein concentration of the eluant was determined using an in-line Optilab T-rEX Interferometric Refractometer (Wyatt Technology Corp.). The weight-averaged molar mass of species within defined chromatographic peaks was calculated using the ASTRA software version 6.0 (Wyatt Technology Corp.), by construction of Debye plots (KC/*R*θ versus sin^2^[θ/2]) at 1 s data intervals. The weight-averaged molar mass was then calculated at each point of the chromatographic trace from the Debye plot intercept and an overall average molar mass was calculated by averaging across the peak.

### Sedimentation velocity analytical ultracentrifugation (SV-AUC)

Sedimentation velocity analytical ultracentrifugation (SV-AUC) experiments were performed at 20°C with an XL-A analytical ultracentrifuge (Beckman-Coulter, Fullerton, CA, USA) and a TiAn60 rotor with two-channel charcoal-filled epon centerpieces and quartz windows. Experiments were performed in 20 mM HEPES–NaOH pH 7.5, 300 mM NaCl and 0.1 mM TCEP at concentrations of 0.16–1.0 mg/ml. Complete sedimentation velocity profiles were collected at 280 nm every 30 s for 200 boundaries at 40 000 rpm. Data were fit using the *c*(*s*) distribution variant of the Lamm equation model, as implemented in the program SEDFIT (27). After optimizing meniscus position and fitting limits, the sedimentation coefficient(s) and best-fit frictional ratio (*f/f*_0_) was determined by iterative least squares analysis. Sedimentation coefficients were corrected to *s*_20,w_ based on the calculated solvent density (ρ) and viscosity (η) derived from chemical composition by the program SEDNTERP (Sedimentation Utility Software. Hayes *et al.*, Amgen Corp., http://www.rasmb.bbri.org/).

### Sedimentation equilibrium analytical ultracentrifugation (SE-AUC)

Analytical ultracentrifugation experiments were performed with an XL-A analytical ultracentrifuge (Beckman–Coulter) and a TiAn60 rotor with six channel charcoal-filled epon centerpieces and quartz windows. SE data were collected at 4°C with detection at 280 nm for 1–3 sample concentrations in 20 mM Tris-HCl pH7.4, 200 mM NaCl, 5 mM DTT. Analyses were carried out using global fits to data acquired at multiple speeds for each concentration with implicit mass conservation using the program SEDPHAT (74). Error estimates for equilibrium constants were determined from a 1000-iteration Monte Carlo simulation. The partial specific volume ($\bar{\upsilon}$), solvent density (ρ) and viscosity (η) were derived from chemical composition by SEDNTERP.

### Size-exclusion chromatography in-line with small-angle X-ray scattering (SEC-SAXS)

Data were collected at the SIBYLS beamline of the Advanced Light Source Light Source II (Berkeley, CA). Data were collected at a wavelength of 1.0 Å in a three-camera conformation, yielded accessible scattering angle where 0.006 < *q* < 3.0 Å^–1^, where q is the momentum transfer, defined as *q* = 4π sin(θ)/ λ, where λ is the X-ray wavelength and 2θ is the scattering angle; data to *q* < 0.5 Å^–1^ were used in subsequent analyses. 100 μl of 2.5 mg/ml dmSMN•G2 or 10 mg/ml MBP-dmSMN^189–220^ were injected and eluted isocratically from a Shodex 804 sizing column equilibrated in 20 mM N/KPO4 pH 7.0, 300 mM NaCl and 1 mM DTT, at room temperature. Eluent from the column flowed into a 1 mm capillary for subsequent X-ray exposures at 1-s intervals. Plots of intensity from the forward scatter closely correlated to in-line UV and refractive index (RI) measurements.

### Singular Value Decomposition with Evolving Factor Analysis (SVD-EFA)

SVD-EFA analysis of the SEC-SAXS data sets were performed as previously described (78), as implemented in the program RAW (77). Buffer subtracted profiles were analyzed by singular value decomposition (SVD) and the ranges of overlapping peak data determined using evolving factor analysis (EFA). The determined peak windows were used to identify the basis vectors for each component and the corresponding SAXS profiles were calculated. When fitting manually, the maximum diameter of the particle (*D*_max_) was incrementally adjusted in GNOM (79) to maximize the goodness-of-fit parameter, to minimize the discrepancy between the fit and the experimental data, and to optimize the visual qualities of the distribution profile. The theoretical SAXS profiles for atomic models were created using the FoxS program (80). The models were rendered using the program PYMOL (73).

### Small-angle X-ray scattering (SAXS)

Sample scattering profiles from beam line X21 at the National Synchrotron Light Source (NSLS, Upton, NY, USA) were collected with a MAR 165 CCD detector (MAR USA, Inc., Evanston, IL). Two-dimensional images were integrated using software developed at the beam line into 1D intensity profiles as a function of *q*. Measurements were taken at 20°C with a sample-to-detector distance of 835 mm and an X-ray wavelength of 1.239 Å; scattering profiles covered a q range from 0.009 to 0.45 Å^–1^. The sample holder was a 1-mm quartz capillary (Hampton Research, Aliso Viejo, CA) that was sealed across the evacuated beam path. Both ends of the capillary were open to allow the sample to flow continuously through to minimize radiation damage to the sample. Each measurement required 30 μl of sample for 30 and 60 s exposure times. After each measurement, the capillary was washed repeatedly with buffer solution and purged with compressed nitrogen.

### Parallel axis theorem analysis (56)

Knowing the individual radii of gyration (*R*_1g_ and *R*_2g_) of two objects and their overall *R*_g_ as a complex, the distance r between the two bodies can be expressed as such:

$$\begin{equation*}{\rm{\;}}{R_{\rm g}}^2 = {f_1}\;{R_{1{\rm g}}}^2 + {f_2}{R_{2{\rm g}}}^2 + {f_1}{f_2}{L^2}\end{equation*}$$

where

$$\begin{equation*}{f_i} = \frac{{\smallint {\rho _i}{\rm d}{V_i}}}{{\smallint {\rho _i}{\rm d}{V_1} + \;\smallint {\rho _i}{\rm d}{V_2}}}\,(i = 1,2)\end{equation*}$$

These are the relative scattering components of the two particles, with respective scattering-length densities ${\rho _i}$ and volumes ${V_i}$.

### Mixed oligomer experiments

The SMNΔ5•Gemin2 complex was expressed and purified as described above, with an additional Mono Q ion exchange purification step. HFS-SMNΔ5•Gemin2 (wild-type, patient mutation variants, and a truncated SMNΔ5^14–156^ negative control) was expressed using a pColADuet-derived vector and purified in the same way. Using both expression vectors, the two complexes were also co-expressed and purified using the same Ni-NTA, Chitin Binding Resin. The final material was spin-concentrated in a final buffer of 20 mM HEPES-NaOH pH 7.5, 400 mM NaCl and 10 mM DTT. The recovered proteins were analyzed by 12.5% SDS-polyacrylamide gel electrophoresis and visualized using acidic Coomassie blue.

For mixing experiments, material was further purified using Mono Q ion exchange purification steps. Protein mixtures were nutated at 10 μM concentrations for one hour at 25°C or 4°C before binding to 0.5 ml of Ni-NTA resin at room temperature. Resin was washed twice with ten column volumes of wash buffer (20 mM Na/KPO4 pH 7.4, 400 mM NaCl, 20 mM imidazole) before elution with 200 mM imidazole and subsequent SDS-PAGE analysis.

### Yeast complementation analysis

A haploid *Schizosaccharomyces pombe* strain (spGV40) with chromosomal *smn1+* replaced by a kanMX6 marker and episomal *smn1+* provided on a *ura4+* plasmid (pGV2887) was constructed using standard methods (28) from strain ATCC 96116 (*h+ his3-D1 leu1-32 ura4-D18 ade6-M210*). pGV2887 was constructed by insertion of a genomic PCR fragment extending ∼500 bp upstream and downstream of the Smn1p coding sequence into the BamHI/SalI sites of pUR19 (29) using primers 5′-gtcagt-ggatccttactgagaagtcctcgctaaacc-3′ and 5′-gtcagtgtcgacatcaccaccgtggagacgaac-3′. Wild-type and mutant Smn1p coding sequences were cloned into pREP3X (30) and transformed into spGV40 with selection on Edinburgh minimal medium (Bio 101) supplemented with histidine, adenine, lysine, and thiamine but not leucine (EMMT-Leu). Six transformants were patched onto EMMT-Leu plates and replica plated to dilution on successive EMMT-Leu plates followed by FOA selection plates containing 0.5% yeast extract, 80 μg/ml adenine sulfate, 3% dextrose, 0.5 mg/ml 5-fluoroorotic acid (Research Products International), adjusted to pH 4 with acetic acid. Growth on FOA medium requires loss of the ura4+ marker, indicating that the pREP3X plasmid can support growth in the absence of *smn1+* from pGV2887. Experiments were repeated three or more times with consistent results for all constructs but the Y137H and Y137R mutants, which sometimes displayed more growth on FOA than shown in Figure 3B, but always much less than wild-type Smnp. Basal expression in the presence of thiamine was sufficient for complementation of the *smn1+* deletion.

### Drosophila husbandry, transgenesis and viability

Balanced transgenic fly lines (overall genotype: *Smn^X7^, Flag-Smn^TG^*/TM6B-GFP) were generated as previously described (21), where ‘TG’ denotes a given transgene. Briefly, the lines were generated using ΦC31 integration at an insertion site located in chromosome band position 86F8 and these lines were introgressed into an *Smn^X7^* null mutant background. The *Smn* transgenic construct is a ∼3kb fragment containing the entire *Smn* coding region, expression of which is driven by the native *Smn* promoter. The transgene also contains an N-terminal 3X-FLAG tag. The *Smn^X7^* and *Smn^D^* alleles are previously described null alleles (31,32), and both stable stocks are GFP-balanced. To generate single-copy transgenic mutants (*Smn^X7^,Smn^TG^/Smn^X7^*), virgin *Smn^X7^*/TM6B-GFP females were crossed to *Smn^X7^,Smn^TG^*/TM6B-GFP males. Crosses were performed on molasses-based agar plates with yeast paste, and then GFP-negative larvae were sorted into vials containing standard molasses fly food at the 2^nd^-instar stage to prevent competition from heterozygous siblings.

To measure viability, 25–50 GFP-negative progeny at the late second to early third-instar stages were sorted into vials containing standard molasses fly food. After sufficient time had passed, pupal cases were counted and marked, and any adults were counted and removed from the vial. Any new pupal cases or adults were recorded every two days. The % viability was calculated at both the pupal and adult stages. Pupal viability (% pupation) was calculated by dividing the number of pupal cases by the initial number of larvae and multiplying by 100 (# pupae/# initial larvae × 100). Adult viability (% eclosion) was calculated similarly but using the number of adults as the numerator (# adults/# initial larvae*100).

To assess larval motor function, crosses were maintained and progeny were raised at 25°C. Once the larvae reached the wandering third-instar, 1–5 animals were placed onto the locomotion stage (a large molasses plate) at room temperature. The stage was then placed into a recording chamber to control light and reflections on the stage. Once all larvae were actively crawling, movement was recorded for at least 62 s on an iPhone6 at minimum zoom. Two recordings were taken for each set of larvae. At least 30 larvae were recorded for each experimental group. Locomotion videos were transferred to a PC and converted to raw video .avi files using the ffmpeg program. Videos were then opened in Fiji/ImageJ (https://imagej.net/Fiji), trimmed to about 60 s of video and converted into a series of binary images. The wrMTrck plugin for ImageJ (http://www.phage.dk/plugins/wrmtrck.html) was used to analyze the video and determine larval size, average speed of movement, and average speed normalized to larval size (body lengths per second or BLPS) (33). Each larva was treated as an individual when calculating average and standard error.

### Disulfide crosslinking

The MBP-hsYG box fusion used for crosslinking studies has been described (25). To maintain a cysteine-free background, a C289A background was used for all experiments. A similar construct was prepared for spSMN YG box fusions, where SMN residues 112–152 were fused to the penultimate Thr residue in *Escherichia coli* MBP with a Gly-Ser linker. The fusion proteins were expressed in BL21(DE3) cells and purified using amylose resin (NEB) using the vendor's protocol under reducing conditions. After adjusting the concentrations to 5 μM, the proteins were dialyzed versus 20 mM Tris-HCl pH 7.4, 200 mM NaCl, 1 mM EDTA and 10 mM maltose to remove reducing agent. Protein samples were then treated with either 1 mM DTT or 160 μM freshly prepared diamide for 1 h at 20°C and immediately analyzed by non-reducing SDS-PAGE. Dialysis and crosslinking experiments were repeated three times and the average percent dimer formation was determined following quantitation of Coomassie stained gels. Non-reducing SDS-PAGE of all constructs showed only monomeric MBP-YG fusions following DTT treatment.

### Structural modeling

A structural model of a dmSMN YG box dimer was generated by replacement of side chains in the spSMN YG box (PDB ID 4RG5) with those that differ in the dmSMN sequence. A hsSMN dimer model was extended to include additional N- and C-terminal residues using the same superposition. A model of an hsSMN tetramer was generated by superposition of one helix from each of two YG box dimers with helices forming a right-handed cross in the *E. coli* glycerol facilitator structure (PDB ID 1FX8). Using COOT (34) along with a rotamer library, residues 269–277 of one hsSMN helix were superimposed onto residues 92–100 of 1FX8 and the same residues of a second hsSMN helix were superposed onto residues 14–22 of 1FX8. The resulting interface is tightly packed and required manual adjustment of His273, Tyr277 and Met278 side chains. Further adjustment of the inter-helical distance and angle would be required to fully relieve the remaining steric contacts.

## RESULTS

### Sequence and structural conservation of the SMN YG box

**Figure 1.**
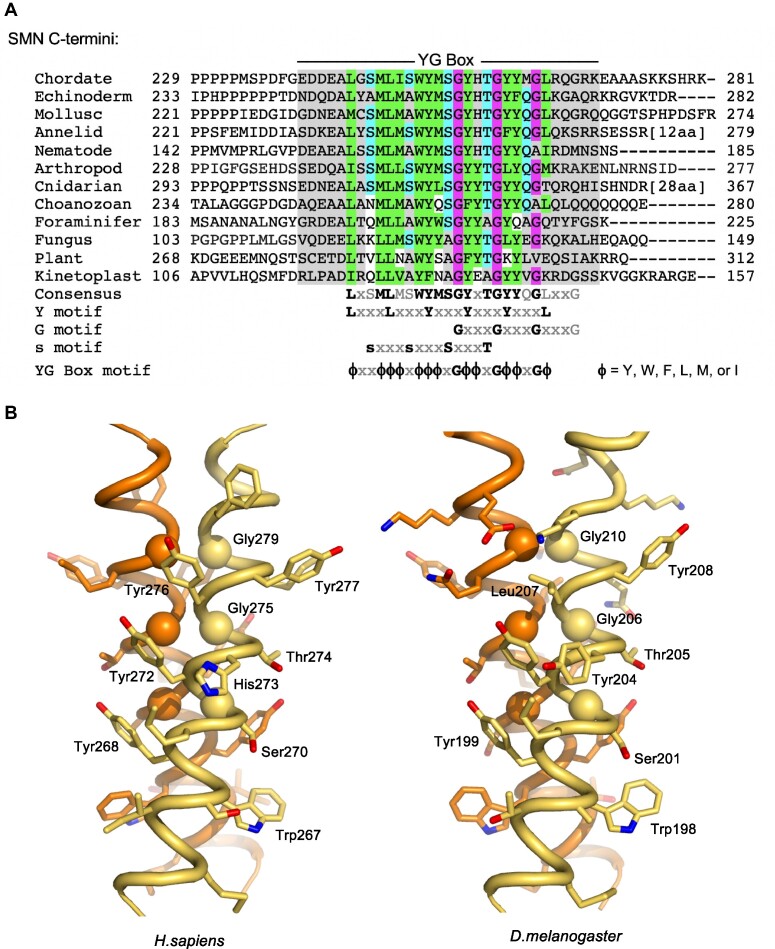
Structural conservation of the SMN YG box domain. (**A**) Phylogenetic analysis of SMN C-termini from a diverse selection of eukaryotes. Conserved glycine residues are shaded in magenta, hydrophobic residues are in green, and polar residues in teal. Note the regularized spacing of residues in three overlapping motifs (Y, G and s) that are contained within the overall YG box consensus. (**B**) Structure of the SMN YG box dimer. Experimental atomic structures of the human (PDB ID: 4GLI) and fission yeast (PDB ID: 4RG5) SMN dimers were used to generate extended models of the human (left) and fruitfly (right) proteins. The three well-conserved glycine residues are shown as Cα spheres. Atomic views were rendered using the program PYMOL (73). See supplemental Supplementary Figure S1 for a more extensive phylogenetic comparison along with additional ultrastructural models.

A phylogenetic comparison of the C-terminal domain of diverse SMN orthologs reveals several highly conserved sequence features. In addition to the overall hydrophobic character of this domain, there are three overlapping motifs (Figure 1A). The G-motif (GxxxGxxxG) is a signature of certain glycine zipper transmembrane proteins that form coiled-coil dimers and oligomers (35). The s-motif denotes the presence of small amino acid residues (typically Ser, Ala, Thr). The Y-motif (YxxxYxxxY), on its own, is common among unstructured regions of tyrosine-rich RNA binding proteins. However, the interdigitation of these highly conserved tyrosine and glycine residues was shown to form a unique structural variant of the glycine zipper motif, termed a YG zipper (25).

Within the SMN YG box dimer structure (Figure 1B), each of the three Y-motif side chains packs against the main chain atoms of the *i* + 3 glycine residue on the opposing helix. Face-to-face apposition of the three glycine residues with their counterparts in the partner helix leads to a more intimate interface between helix backbones than is found in canonical coiled-coil dimers. This resulting network of inter-subunit interactions in SMN is conserved from yeast to human (24,25). The broad conservation of SMN YG box sequence features among other eukaryotic groups (e.g. in *Amoebozoa*, *Plantae*, *Rhizaria* and *Excavata*) strongly supports the importance of these inter-subunit Y-G interactions in dimer formation (Figure 1A and Supplementary Figure S1A). Models of the fruitfly and nematode YG zipper dimers were readily generated from the human and yeast X-ray structures, without any initial steric clashes or need for changes to the main chain helix conformation (Figure 1B and Supplementary Figure S1B).

The SMN•Gemin2 heterodimer (SMN•G2) is thought to be the fundamental structural component of the SMN complex, and this subunit is known to form higher-order multimers *in vitro* (24,25). Human Gemin2 is monomeric, and does not oligomerize, either alone or in the presence of pre-formed SMN•G2 dimers (24). For our purposes here, an oligomer of SMN•G2 refers to a species with a stoichiometry of (SMN•G2)_*n*_, where *n* = 2 for a dimer, *n* = 4 for a tetramer, etc. Notably, purified recombinant SMN (36), as well as those complexes isolated from animal cells (37,38), are of a size that is much larger than that predicted for a dimer. Yet we currently have no structural models for SMN higher-order oligomerization. Thus, understanding the cellular circumstances and molecular mechanisms whereby SMN assembles into higher-order oligomers is crucial.

### Biophysical properties of metazoan SMN•Gemin2 complexes are conserved

*In vitro* and *in vivo*, human SMN forms complexes whose hydrodynamic size is much greater than that predicted from its components (24,25,36). Size-exclusion chromatography coupled with multi-angle light scattering (SEC-MALS), analytical ultracentrifugation (AUC) and small-angle X-ray scattering (SAXS) data have shown that *Homo sapiens* hsSMN•G2 and hsSMNΔexon5 (hsSMNΔ5)•G2 complexes exist within a temperature-dependent tetramer to octamer equilibrium with little presence of dimers (24). For reasons unknown, complexes from fission yeast *S. pombe* (spSMN•G2) exist *in vitro* primarily as dimers and tetramers, with no evidence of octamers, or temperature dependence (24). Furthermore, estimates of SMN protein concentration within living fission yeast cells (39) suggest that the concentration of the dimer (∼15 nM) is far below the measured dissociation constant for the tetramer (∼1 μM) (24). Hence, the spSMN•G2 dimer is likely the most abundant species *in vivo* and is presumed to be the basal functional complex required for cell viability (7).

**Figure 2.**
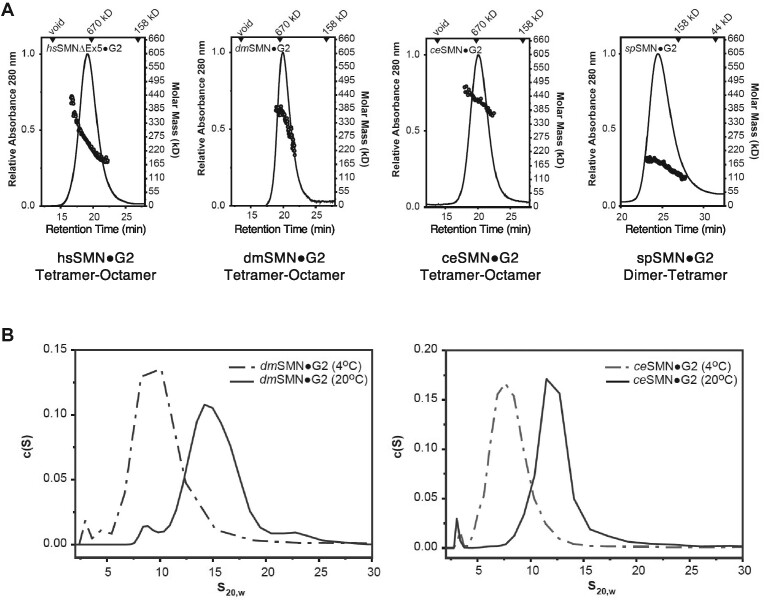
Biophysical properties of wild-type SMN•Gemin2 complexes are conserved. (**A**) SEC-MALS analysis performed at 20°C for *Homo sapiens* (hs), *Drosophila melanogaster* (dm), *Caenorhabditis elegans* (ce) and *Schizosaccharomyces pombe* (sp) SMN•G2 complexes. As previously observed for the human and fission yeast complexes (24,25), SEC elution times suggest the presence of larger multimers in metazoans. However, *M*_w_ values from the in-line light scattering indicate a range of oligomers in each case; the oligomeric range is assigned below each panel. (**B**) Sedimentation velocity (SV-AUC) analyses of dmSMN•G2 (left) and ceSMN•G2 (right). At 25°C, the fruitfly complex sediments as a broad ∼15 S peak but at 4°C a smaller ∼8 S peak is observed. Similarly, the nematode complex sediments as a broad ∼12S peak at 25°C but smaller ∼7S peak at 4°C. See Supplementary Figure S2 for additional biophysical characterization of the dmSMN complex and its components.

To determine whether the biophysical properties of metazoan SMN proteins are conserved, we generated SMN•G2 complexes from the fruitfly *Drosophila melanogaster* (dmSMN•G2) and nematode *Caenorhabditis elegans* (ceSMN•G2) and carried out a variety of biophysical measurements. As shown in Figure 2, SEC-MALS shows that fruitfly and nematode SMN•G2 complexes mirror those of the human complex at room temperature. The weight-averaged molecular masses (*M*_w_) of the complexes derived from MALS (Figure 2A, Supplementary Table S1) are consistent with a tetramer-octamer distribution and the sedimentation velocity (SV) profiles obtained via AUC (Figure 2B) are similar in size and breadth to those previously determined for the hsSMN•G2 (24). The sedimentation coefficients of the human complex were also shown to be temperature-dependent, with a shift towards smaller species observed at 4°C. This behavior was recapitulated with the nematode and fly complexes in SV-AUC analysis (Figure 2B). The temperature dependence of metazoan SMN oligomerization implies that the larger multimers are stabilized primarily by hydrophobic interactions.

The fly complex was distinct among the systems examined in that both dmSMN and dmG2 could be purified separately and the complex reconstituted *in vitro*. In contrast, the human, worm, and yeast systems required bacterial co-expression to obtain soluble wild-type complexes. We used this opportunity to determine the properties of dmSMN and dmG2 alone. As anticipated, dmSMN is oligomeric, with a sedimentation coefficient of 11S, and dmG2 is largely monomeric, with a very modest dimerization (33.4 μM ± 5) constant, as determined by SE-AUC (Supplementary Figure S2). Previous studies with hsSMN and spSMN showed that the oligomeric behavior of the SMN•G2 complexes was recapitulated by fusions of the maltose binding protein (MBP) to the SMN YG boxes (24,25). This behavior is true of the fly complex as well; MBP-dmSMN^186–220^ forms complexes spanning the tetramer-octamer range (Supplementary Figure S2). As a final test of the fruitfly proteins, we analyzed dmSMN•G2 complexes and MBP-dmSMN^186–220^ fusions using size-exclusion chromatography in-line with synchrotron small-angle X-ray scattering (SEC-SAXS). The scattering profiles for the dmSMN•G2 complex were deconvoluted into two primary components: tetramers and octamers (Supplementary Figure S2, Supplementary Table S3). Thus, as analyzed by AUC, SEC-MALS and SEC-SAXS at micromolar concentrations, metazoan SMN•G2 complexes exist as a mixture of oligomers spanning tetramers to octamers, whereas the fission yeast complex forms dimers and tetramers.

### Genetic and biophysical analysis of SMN mutations in *S. pombe*

**Figure 3.**
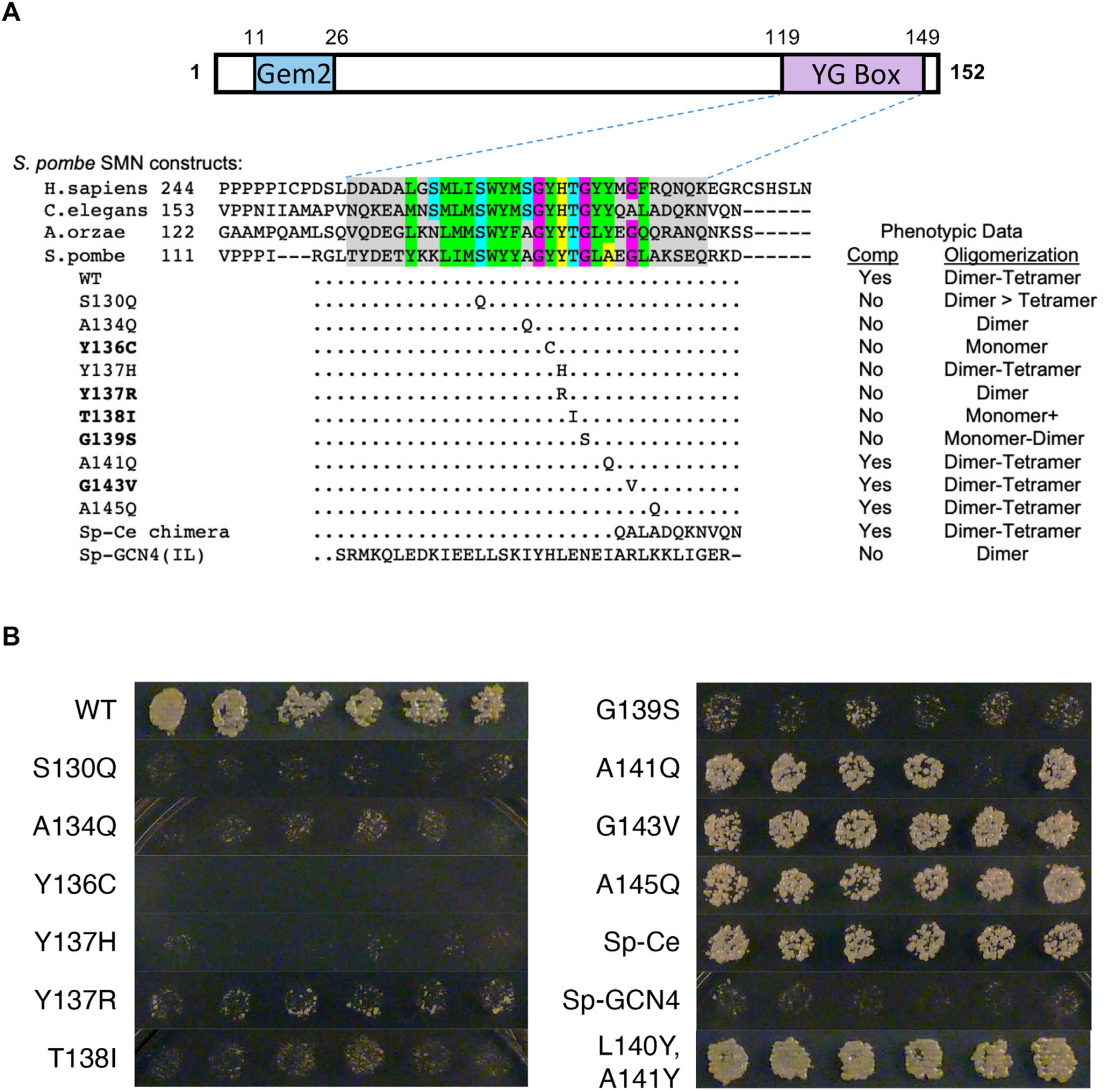
Genetic and biophysical characterization of fission yeast SMN mutants. (**A**) Cartoon of *sp*SMN protein, showing relative location of the Gemin2 (Gem2) binding domain and the YG box. An alignment of YG box sequences from the human (*H. sapiens*), nematode (*C. elegans*), köji mold (*A.orzae*) and fission yeast (*S. pombe*) SMN orthologs is shown for comparison. Genetic complementation analysis of a fission yeast *smn1* null allele was performed with a wild-type (WT) rescue construct or with a variety of chimeric or point substitution mutation constructs. Mutants that correspond to human SMA-causing missense alleles are shown in bold text. Ability to complement (Comp) the growth defect observed in the null mutant background is indicated. Recombinant SMN•G2 complexes of these same mutant constructs were generated *in vitro* and subjected to SEC-MALS and SE-AUC analysis, as described in Figure 2. The range of oligomeric species detected by SEC-MALS is also indicated. See Supplementary Table S2 for additional details regarding biophysical characterization of spSMN complexes. (**B**) Complementation of *smn1+* deletion in *S. pombe*. Constructs expressing *smn1* variants under control of the *nmt1* promoter were transformed into haploid *S. pombe* containing a deletion of chromosomal *smn1+* and episomal *smn1* expressed from a *ura4+* plasmid. Individual transformants were patched onto minimal medium containing thiamine, then replica plated to dilution on selection plates containing 5-fluoroorotic acid (5-FOA). Strong growth on FOA medium requires loss of the *ura4+* plasmid, and therefore complementation of *smn1Δ* by the *smn* construct.

Although *Smn* has been lost from the budding yeast genome during evolution, it is an essential gene in the fission yeast (40–42). Fungal SMN proteins contain only two of the three conserved domains, the Gemin2 binding region and the YG box (Figure 3A), both of which are essential for viability (42). To understand the effects of YG box missense mutations on cell growth and SMN oligomerization potential, we carried out site-specific mutagenesis and compared phenotypes of the mutant proteins when expressed *in vivo* and *in vitro*. As summarized in Figure 3, expression of the wild-type Smn1p construct fully complemented deletion of the endogenous *smn1* gene. In contrast, most of the mutant proteins failed to complement growth *in vivo* and were likewise affected in their ability to form SMN•G2 tetramers when expressed *in vitro* (Figure 3). For example, replacement of three of the conserved ‘s-motif’ residues (Figure 1A) with bulky residues like glutamine (S130Q, A134Q) or isoleucine (T138I) all caused defects in oligomerization and cell growth (Figure 3). Substitution of other YG box alanines with glutamine (A141Q or A145Q) had little effect on growth or oligomerization.

As expected, modeling of human SMA patient-derived missense alleles in *S. pombe* revealed that mutations of highly conserved YG box residues (Y136C, Y137R, T138I and G139S) failed to complement the *smn1* deletion (Figure 3). Moreover, all four of these mutations caused defects in SMN oligomerization to different extents (Figure 3A). The spY136C protein is monomeric, consistent with studies of this mutation in other model systems that showed a failure to interact with itself in pulldown assays (e.g. hsY272C and dmY203C (15,21)). We note that one of the SMA-causing alleles (spG143V) did not display a defect in complementation (Figure 3A, B). Interestingly, overexpression of this same spG143V mutant protein in a wild-type *smn1* background was previously shown to impede growth of *S. pomb*e in a dominant negative fashion (41), but here we show that this allele is viable when expressed in the absence of the wild-type protein (Figure 3B). Furthermore, and similar to previous findings in *Drosophila* S2 cells (dmG210V, (21)) and MBP-hsSMN^252–294^ fusion constructs (hsG279V, (25)), we show that the spG143V construct is able to form higher-order oligomers (Figure 3).

Given that this C-terminal glycine (hsG279) is the least well-conserved position among the three G-motif residues (see Supplementary Figure S1A), the spG143V result suggests that the observed metazoan SMA phenotype (human: Type I, (43); fruitfly: Class 2, (44)) is caused by a distinct mechanism-of-action. Indeed, the C-terminus of the nematode SMN orthologue diverges in this region and a chimeric fusion of the yeast and worm YG boxes fully complements the *smn1* null mutation *in vivo* and displays an oligomerization profile that is very similar to that of the wild-type *S. pombe* protein *in vitr*o (Figure 3A). Thus, we conclude that the C-terminal G-motif glycine is not required for SMN self-interaction.

In order to determine whether dimerization alone is sufficient for SMN to carry out its functions in fission yeast, we generated a series of chimeric fusion constructs that replace the entire *S. pombe* YG box with a leucine zipper domain derived from the *S. cerevisiae* transcriptional activator, GCN4. The ∼34 a.a. coiled-coil motif present within GCN4 has been thoroughly studied and can be tuned to generate dimers, trimers or higher-order oligomers (45). When fused to spSMN, the GCN4(IL) peptide (Sp-GCN4 chimera, Figure 3A, Supplementary Table S1) forms obligate dimers when analyzed by SEC-MALS, SV-AUC and SAXS, whereas the GCN4(LI) and (II) fusion proteins form multiple higher-order oligomeric species (Supplementary Tables S1, S2, S4, and Supplementary Figure S6). Importantly, all three of these chimeric proteins fail to complement the *smn1* deletion *in vivo*. This result was not unexpected, as the YG box is thought to provide binding surfaces for Sm protein substrates as well as other members of the SMN complex (6,19). These data clearly show that C-terminal multimerization, *per se*, is insufficient to support organismal viability.

### Oligomeric properties of human SMN•Gemin2 complexes bearing SMA-causing YG box point mutations

**Figure 4.**
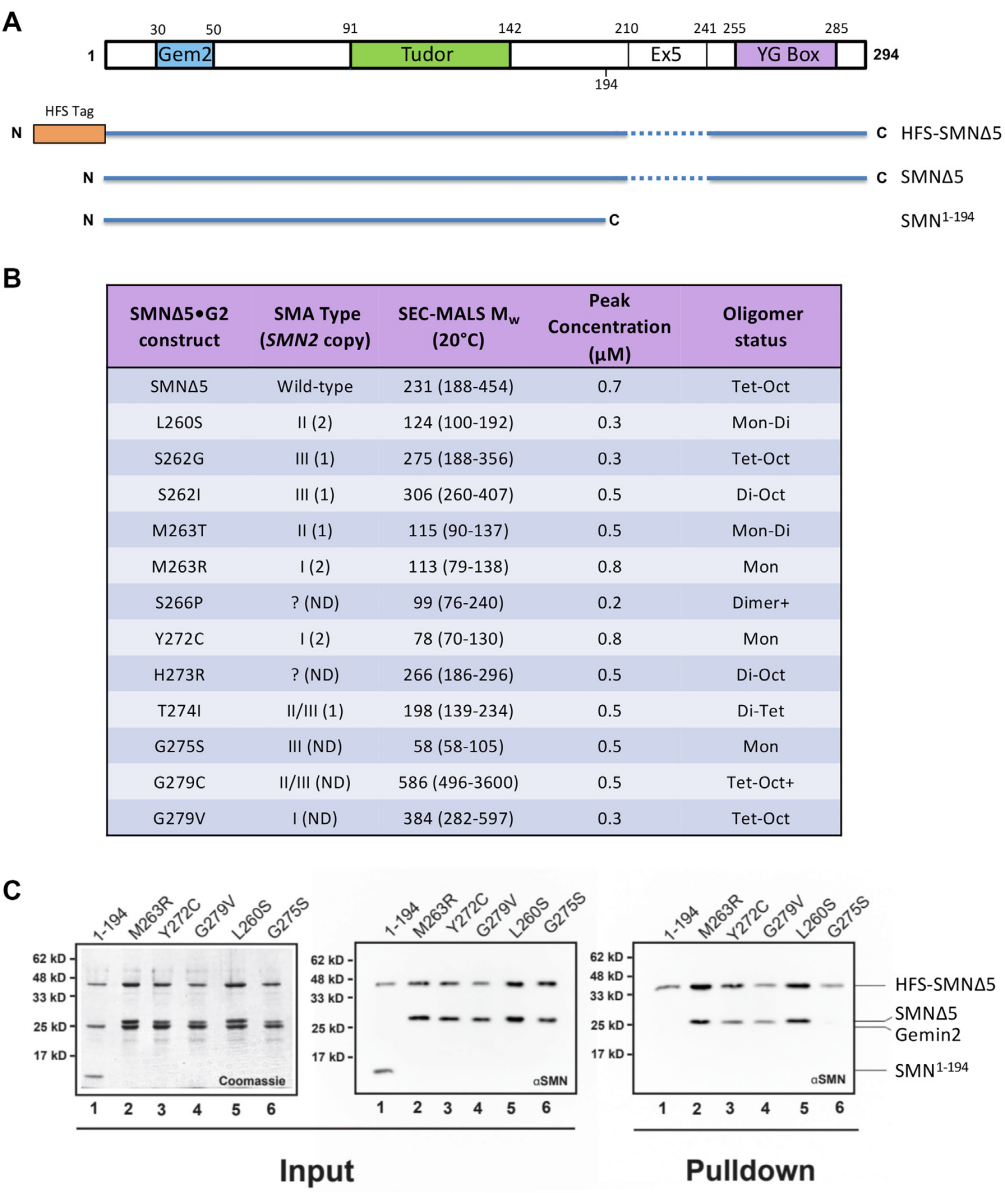
Biophysical characterization of human SMN•Gemin2 complexes bearing SMA-causing YG box missense mutations. (**A**) Cartoon of hsSMN protein, showing the conserved YG box, Gemin2 binding (Gem2) and Tudor domains, along with location of exon5 (Ex5) sequences deleted in the SMNΔ5 construct. (**B**) SEC-MALS analysis of SMA-causing point mutant constructs in the hsSMNΔ5•Gemin2 backbone. All of the mutations were generated on the SMNΔ5 backbone. Additional details regarding reported human *SMN2* copy number and SMA patient phenotype are also provided (22). (**C**) Formation of mixed oligomers between wild-type hsSMN and SMA patient mutations *in vitro*. A subset of the patient-derived mutations was screened for the ability to form mixed oligomers with wild-type SMNΔ5. The left two panels show Coomassie and western blot analyses of SDS-PAGE gels of the input material following bacterial co-expression and lysate clarification; the last panel shows a western blot of the resulting pulldown using Chitin-binding resin. As a negative control, the ability of a truncated SMN lacking the YG oligomerization domain (SMN^1–194^) was also assayed. After co-expression and elution from the chitin binding resin, four of the five patient mutant samples (M263R, Y272C, G279V and L260S) demonstrated the ability to form mixed oligomers. Among these five missense constructs, only SMNΔ5(G275S) failed to co-purify with wild-type SMNΔ5.

Thus far, we have only analyzed the oligomerization status of human SMA-causing YG box missense mutations in the context of MBP-fusion constructs (25). To directly compare the molecular size data in Figure 3B, we generated hsSMNΔ5•G2 complexes that contain SMN point mutations and analyzed their properties using SEC-MALS (Figure 4). Exon 5 encodes a proline-rich region in hsSMN that causes the SMN•G2 complex to have reduced solubility, making biochemical analyses difficult. We therefore used the naturally occurring SMNΔ5 isoform for these experiments. As shown in Figure 4A, hsSMN and hsSMNΔ5 contain all three conserved domains.

For the most part, the severity of the SMA phenotype correlates well with the oligomerization potential of the corresponding SMN•G2 complexes, and with the previously reported oligomerization status of the corresponding MBP-fusion constructs (Figure 4B; (25)). Several of the SMA point mutants are within a strongly conserved hydrophobic region that precedes the Y, G and s motifs (Figure 1A). For example, L260S, M263T and M263R would be expected to weaken or disrupt the helical dimer shown in Figure 1B and that is what we observe. Although the crystal structure of the hsSMN YG box (25) did not provide insights into much of this region, the longer N-terminal extension present in the spSMN YG box structure (24) indicates clear roles for these residues in extending the hydrophobic interface between helices.

In certain cases, the SMA-causing mutations have little effect on the oligomeric state of SMN•G2. Substitutions at human Ser262, His273 and Gly279 each result in complexes that can form higher-order oligomers. The hsG279V mutant is particularly interesting because one might expect that a bulky valine substitution would be incompatible with the close Gly–Gly contacts present in the YG box dimer. However, as discussed above for the equivalent yeast substitution (spG143V), the SMN dimer apparently tolerates a larger separation of helices near hsGly279, and the resulting structural perturbations do not affect formation of higher-order oligomers.

The experiments summarized in Figure 4B address the homo-oligomerization potential of SMA-causing point mutations but do not indicate whether the mutant proteins are likely to form hetero-oligomers with wild-type SMN. To test this idea, we co-expressed a subset of the more severe SMA alleles with hexahistidine-FLAG-SUMO (HFS)-tagged SMNΔ5 and Gemin2, purified the complexes, and determined whether the untagged mutant proteins copurified with Ni-NTA beads (Figure 4C). The M263R, Y272C, G279V and L260S mutants can each interact with wild-type SMN and might therefore be expected to exhibit dominant negative phenotypes *in vivo*. Consistent with these observations, the dmM194R and dmY203C mutants display dominant phenotypes over the maternal SMN contribution in the fly (21) and spY136C suppresses growth when co-expressed in fission yeast (41).

The hsG275S mutant does not interact with wild-type SMN (Figure 4C). On a structural level, this is not a surprising finding, given that Gly275 is the central glycine in the YG box and even a single substitution in the interacting Gly–Gly pair in a dimer should be severely disruptive (Figure 1B). This finding provides a compelling explanation for the relatively mild SMA severity of the G275S mutation in humans (Figure 4B), especially given that it renders SMN monomeric. The relative lack of a dominant negative effect also explains why the corresponding dmG206S mutant displays a slightly milder phenotype in the fly as compared to dmY203C (44).

### Genetic and biophysical analysis of YG box mutations in *D. melanogaster*

**Figure 5.**
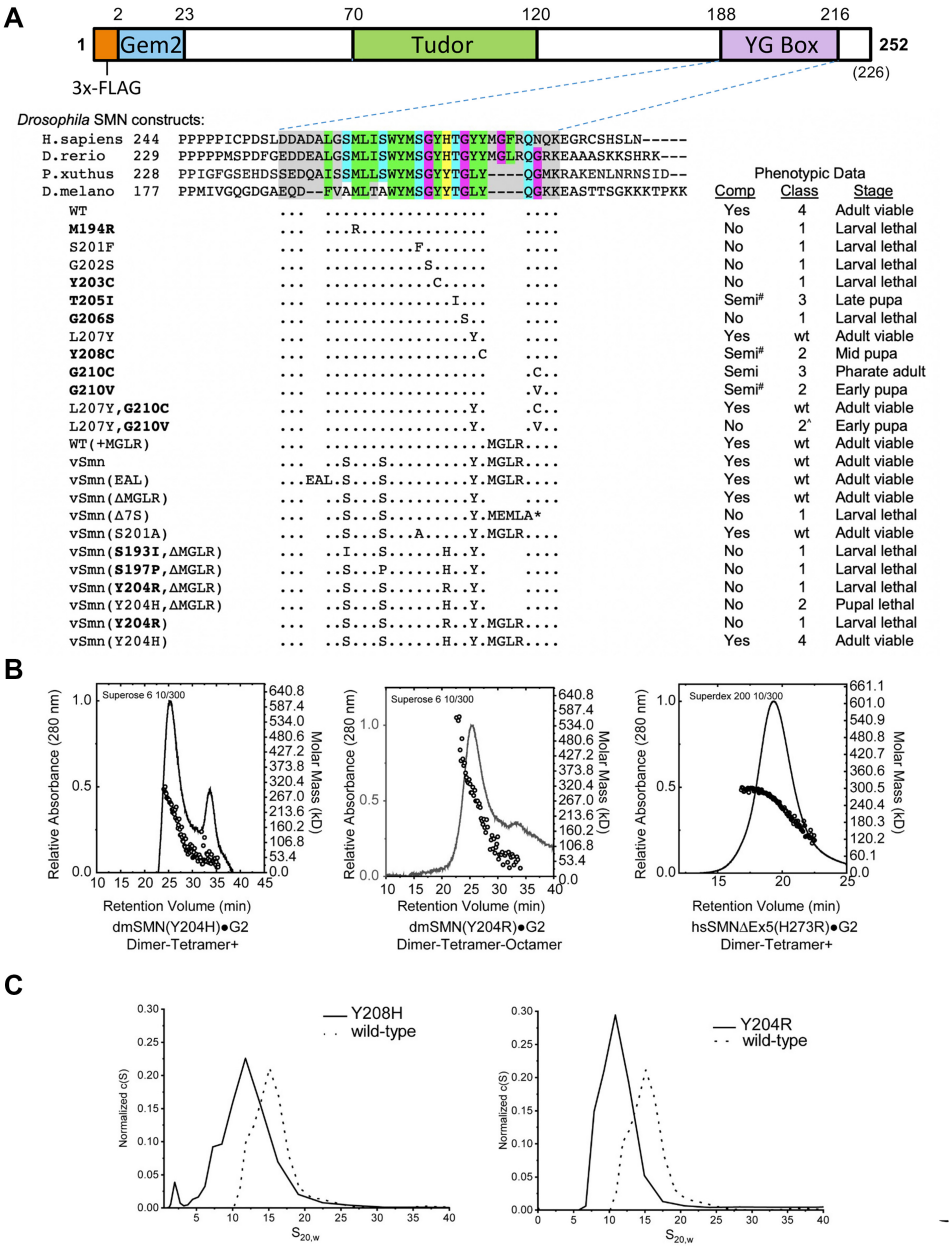
Genetic and biophysical characterization of *Smn* YG box mutations in *Drosophila*. (**A**) Cartoon of the *dm*SMN protein, showing the conserved YG box, Gemin2 binding (Gem2) and Tudor domains, along with the location of the 3x-FLAG tag used for transgenic rescue experiments. An alignment of YG box sequences from the human (*H.sapiens*), zebrafish (*D.rerio*), butterfly (*P.xuthus*) and fruitfly (*D.melanogaster*) SMN orthologs is shown for reference. Phenotypic comparisons of an extensive panel of *Drosophila* YG box substitution mutations are summarized below the alignment. With the exception of S201F and G202S, which are point mutations in the endogenous *Smn* gene (55), the rest of the panel is comprised of transgenic constructs that have been recombined with an *Smn* null allele ((21); this work). Genetic complementation analysis (Comp) was performed using either a wild-type (WT) or mutant *Flag-Smn* transgene. Mutants that correspond to human SMA-causing missense alleles are shown in **bold** text; the classification system used for fly SMA models was described previously (44). See Supplementary Figure S4 for pupal and adult viability analysis of the fifteen new transgenic fly lines used in this work. Mutant lines that eclose at low frequency (adult escapers) are considered semi-viable; those marked with a # sign are incapable of establishing an independent breeding stock. Class 2^ denotes animals that pupate but display a significant advancement in lethal phase or reduction in eclosion frequency. The asterisk * = stop codon. (**B**) SEC-MALS analysis of SMN•G2 complexes containing mutations at hsH273 (H273R) or dmY204 (Y204H and Y204R) aimed at modeling an SMA-causing missense allele in human *SMN1*. (**C**) Analytical ultracentrifugation of *Drosophila* SMN•G2 complexes. *c*(*S*) sedimentation distributions of wild-type (WT) or mutant (Y204H and Y204R) are shown. Note, biophysical analyses of additional *Drosophila* SMN constructs are presented in Table 1 and Supplementary Figure S2.

As shown in Figure 5A, the overall structure of *Drosophila* SMN is similar to that of the human protein (Figure 4A), and a homozygous null mutation of the endogenous *Smn* gene can be rescued by transgenic expression of a wild-type (WT) FLAG-tagged construct (46). Previously, we showed that expression of disease-causing missense mutations in all three conserved SMN subdomains recapitulates the full spectrum of phenotypic severity observed in human SMA (21,44). For example, the *Drosophila* M194R, Y203C and G206S mutants are severe Class 1 alleles that die during larval stages, whereas Y208C, G210C/V (Class 2) and T205I (Class 3) display intermediate phenotypes that manifest during pupal or adult stages (Figure 5A). Phenotypic comparison between SMA patient mutations and the corresponding animal model is complicated by human *SMN2* copy number variation (14). In cases where the *SMN2* copy number is known, the severity observed in each fly mutant Class is well aligned with that of human SMA patient Type (44).

Phenotypic differences can be illuminating. For instance, we note that the Tudor domain mutants hsI116F/dmI93F and hsW92S/dmF70S are Type I SMA mutations in humans (47,48) but display milder Class 3 phenotypes in flies (21). Interestingly, we recently found that these two fly alleles are highly temperature-sensitive, and become differentially more severe (Class 2^^^) when the mutants are reared at elevated temperatures closer to those of mammals (49). Among the few discordant YG box mutants, the hsG275S (Type III) and dmG206S (Class 1) pair is interesting because, as discussed above, the hsG275S complex is monomeric (Table 1). Although *SMN2* copy number was not determined in the single reported patient bearing this *SMN1* mutation (50), one would have expected a much stronger phenotype. We therefore generated the corresponding SMN•G2 complex (dmG206S) and found that it too is primarily monomeric, as measured by SEC-MALS. Thus, the biophysical properties of recombinant dmG206S (Table 1) are consistent with those of hsG275S as well as with results of GST-pulldown assays in *Drosophila* S2 cells (21).

**Table 1.** Oligomeric properties of SMN•G2 variants

SMN•G2 construct	AUC S_20,w_ (25°C)	SEC-MALS *M*_w_ (20°C)	Peak conc. (μM)	Oligomer status
*S. pombe^#^*	6.5S	∼150 kD (110–180)	2.3	Di-Tet
L140Y,A141Y	n.d.	∼257 kD (152–362)	1.7	Tet-Oct
*C. elegans*	13S	∼326 kD (250–450)	0.2	Tet-Oct
Ce2-184-Sp140-152	n.d.	∼137 kD (137–610)	1.0	Dimer
*D. melanogaster*	14S	∼345 kD (202–380)	0.4	Tet-Oct
Y204H	12S	∼261 kD (154-295)	0.2	Di > Tet-Oct
Y204R	11S	∼295 kD (103-457)	0.1	Di >> Oct+
G206S	4-8S	∼43 kD (42-55)	2.8	Monomer+
Y208A	9S	∼127 kD (66-137)	1.9	Di > Tet
*H. sapiens^#^*	12S	∼247 kD (200-600)	0.14	Tet-Oct
SMNΔ5	12S	∼231 kD (188-454)	0.7	Tet-Oct
SMNΔ7^#^	2S	∼66 kD (58-74)	0.2	Monomer
Y277A	n.d.	∼171 kD (110-220)	0.03	Di > Tet
Hs^1-275^-GCN4(IL)	n.d.	∼113 kD (80-113)	0.3	Dimer
Hs^1-275^-Sp^140-152^	n.d.	∼128 kD (88-227)	2.3	Di > Tet
Hs^1-279^-Sp^144-152^	n.d.	∼285 kD (119-640)	0.1	Di < Tet-Oct

^#^Note: These data from Gupta *et al.* (Ref. 24).

Unknown *SMN2* copy number also hinders interpretation of the SMA phenotype at Gly279. Mutation of this residue to Cys (hsG279C) results in mild Type II/III SMA (51), whereas a Val substitution (hsG279V) causes severe Type I SMA (43). In flies, the corresponding mutations are dmG210C and dmG210V, which were designated as Class 3 and Class 2 alleles, respectively (44). As mentioned above (Figure 3), the fission yeast spG143V mutant is viable. The model in Figure 1B shows that human Gly279 is tightly packed against Tyr276, whereas in flies and yeast the tyrosine is replaced by leucine. In the fly model, Gly210 is tucked into a hydrophobic pocket created by Leu207 and the aliphatic side chain of Lys211 (Figure 1B), and so leucine is predicted to be more tolerant of a valine substitution at Gly210 than is tyrosine. To test this prediction, we generated single and double substitutions at Leu207 and Gly210 in the fly. As expected, the dmL207Y controls are fully viable, whereas a small, but reproducible, fraction of dmG210V mutants can complete development (Figure 5A; (44)). By contrast, the dmL207Y,G210V double mutants display a phenotype that is more severe than dmG210V alone and are completely inviable, with no eclosing adults (Supplementary Figure S4A). On the other hand, the dmL207Y-G210C animals display a phenotype that is less severe than dmG210C alone and are viable (Figure 5A, Supplementary Figure S4A). The results show that Leu207 is indeed more tolerant of a G210V substitution than is Tyr207.

As an animal model, *Drosophila* arguably provides a better overall indication of SMN activity *in vivo* because mutant proteins can be expressed and analyzed in the absence of wild-type SMN (21,44). However, due to sequence differences between the vertebrate and insect YG boxes, certain SMA-causing point mutations could not be effectively modeled using the WT *Drosophila* backbone (Figure 5A). Phylogenetic comparison of the YG box domains from nine different vertebrate clades (see Supplementary Figure S3A) suggests that there has been a deletion of four residues in the C-terminal region of the fruitfly YG box relative to the human sequence (residues _278_MGFR_281_). Among these four residues, Phe280 is a clear phylogenetic outlier, as this residue is typically a leucine (Supplementary Figure S3A). We therefore inserted a sequence encoding MGLR at the corresponding position in fly SMN to create the WT(+MGLR) strain. As shown in Figure 5A, this allele is fully viable. Indeed, these animals eclose at significantly higher frequencies than do those of the WT rescue strain (Supplementary Figure S4B).

In addition, the N-terminal half of the human YG box contains several other sequence differences, notably at conserved s-motif or Y-motif positions (Supplementary Figure S3A). Therefore, we generated additional vertebrate-like transgenic rescue lines (vSmn, vSmn^EAL^, and vSmn^ΔMGLR^) to serve as baseline controls. As summarized in Figure 5A, each of these three fly strains is fully viable, demonstrating that neither the insertion of the MGLR residues nor substitution of Ser for Ala, or Leu for Phe at the other positions has any negative effects on organismal viability. Once again, these *vSmn* strains are healthier overall than the WT rescue line (see Supplementary Figure S4 for details). Also as expected, truncation of the fly protein in this region with MEMLA* to model SMNΔ7, the predominant human *SMN2* gene product, caused early larval lethality (Figure 5A, vSmnΔ7S).

However, when we attempted to model one particular SMA patient-derived mutation (hsH273R, Figure 4B), substitution of the corresponding human wild-type residue into the fly protein (vSmn^Y204H,ΔMGLR^) resulted in complete pupal lethality (Figure 5A, Supplementary Figure S4B). Mutation of this residue to model the disease-causing allele (vSmn^Y204R,ΔMGLR^) significantly worsened the phenotype (Figure 5A, Supplementary Figure S4B). Consistent with these findings, fission yeast spY137H and Y137R mutants failed to complement and displayed an impaired growth defect (Figure 3A). Interestingly, spY137R was entirely defective in formation of higher-order SMN oligomers (Figure 3A), whereas hsH273R was only partially impaired (Figure 4B). We therefore generated dmY204H and dmY204R constructs and analyzed their solution properties in complexes with Gemin2, as described above. As shown in Figure 5B, these constructs both show a significant shift toward dimers. The trace for dmY204H is biphasic, with a clear peak in the monomer-dimer range, whereas the mass profile for dmY204R is consistent with the presence of monomers to large aggregates (Figure 5B).

As described previously, hsHis273 sits on the outer side of the helix and makes no direct contacts along the dimer interface (see Figure 1B). So why is the dmY204H/spY137H substitution toxic in flies and yeast? As shown in the crystal structure of the yeast dimer (Supplementary Figure S1B) and in the fly model (Figure 1B), Tyr204 packs against the adjacent Tyr203 residue and helps to buttress the Y-G interaction that forms between Tyr203 and Gly206 on the other helix of the dimer. Histidine is polar and does not make the same hydrophobic contacts, and thus may not function as well in this role (see Discussion). The dmY204R substitution is even more polar and destabilizing, affecting both dimerization as well as formation of higher-order oligomers (Figure 5B). Interestingly, insertion of the vertebrate MGLR helix extension completely suppresses the toxicity of the histidine substitution (Figure 5A, compare vSmn^Y204H^ to vSmn^Y204H,ΔMGLR^), whereas the SMA-causing arginine substitution remains inviable (compare vSmn^Y204H^ to vSmn^Y204R^). We conclude that the fruitfly YG box is unable to support a histidine at Tyr204, affecting both higher-order multimerization as well as dimerization. In the human system, the hsH273R mutation does not appear to interfere with dimerization; rather, it drives the equilibrium in the direction of lower-order multimers (Figure 5B).

### Formation of higher-order SMN oligomers correlates with metazoan viability

Our detailed structure-function analysis of the YG box has shown that mutations which reduce the oligomerization potential of SMN *in vitro* also tend to have a negative impact on organismal viability *in vivo* (Figures 3–5). However, none of the SMA patient-derived missense mutations analyzed thus far allow dimer formation but prevent assembly of higher-order multimers. We therefore sought to identify a missense mutation that causes metazoan SMN•G2 to form obligate dimers *in vitro* and then determine its phenotype in an animal model. Toward that end, we focused on trying to understand the aforementioned discrepancy between the oligomerization status of wild-type fungal and metazoan SMN complexes.

**Figure 6.**
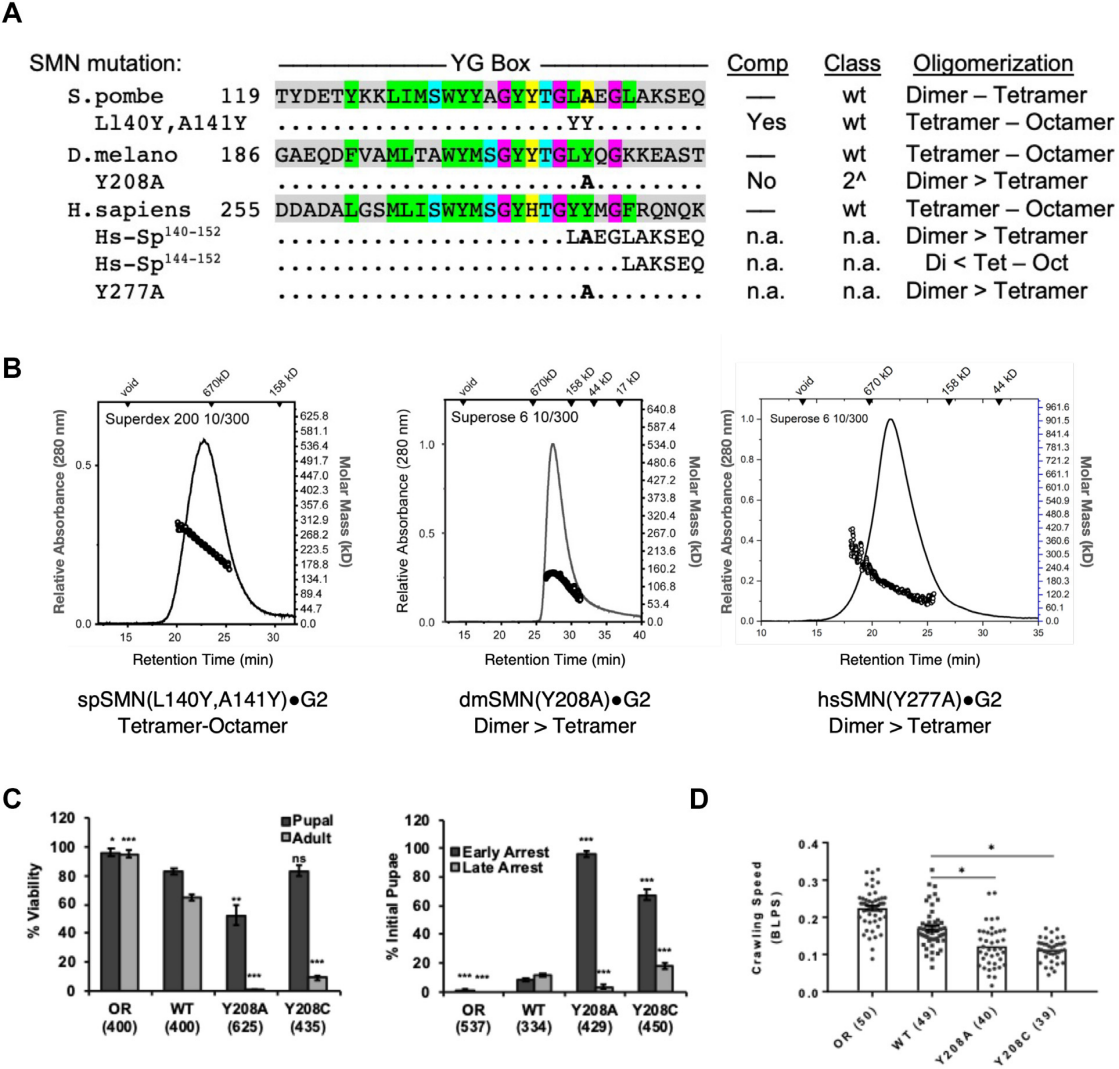
Identification of a specific YG box residue critical for formation of higher-order SMN multimers. A structure-function analysis was carried out in parallel using fission yeast, fruitfly and human SMN. (**A**) Genetic complementation (Comp) and biophysical analyses (Oligomerization) of C-terminal chimeras and point substitution mutants. (**B**) SEC-MALS analysis of SMN•G2 complexes of yeast spSMN^L140Y,A141Y^, dmSMN^Y208A^ and hsSMN^Y277A^. (**C**) Developmental viability of control Oregon Red (OR) flies or animals expressing transgenic Flag-tagged *Smn* wild-type (WT), Y208C or Y208A rescue constructs in the background of an *Smn* null mutation. Left panel: % Viability is the proportion of animals that survive to the pupal (darker gray) or adult (lighter gray) stages, relative to the number of larvae initially collected (n-values in parentheses). Right panel: Breakdown of fraction of animals that arrest during early versus late stages of pupal development. (**D**) Larval locomotion analysis. Average crawling speed, measured in body lengths/sec, in wandering third instar larvae. Data points correspond to measurements of individual larvae. n-values for each genotype are shown in parentheses. Statistical analysis used in panels A–D: Asterisks above the data bars indicate significance versus WT rescue line from one-way ANOVA using the Dunnet correction for multiple comparisons. *P < 0.05, **P < 0.01, ***P < 0.001. *P* > 0.05 is not significant (ns).

As summarized in Table 1, animal SMN•G2 complexes mainly form tetramers and octamers at micromolar concentrations *in vitro*, whereas those of fission yeast exist primarily as dimers along with some tetramers. Chimeric constructs with yeast N-termini and human C-termini (Sp^1–117^-Hs^253–294^) form tetramers and octamers. To better understand this phenomenon, we generated reverse chimeras with C-terminal portions of yeast SMN fused to either human (hs^1–275^-Sp^140–152^) or nematode (Ce^2–184^-Sp^140–152^) N-terminal regions. Interestingly, these complexes were primarily dimeric, although some tetramers were also detected (Table 1). A fusion bearing only the distal C-terminus of the yeast protein (hs^1–279^-Sp^144–152^) restored the ability of the human chimera to form octamers (Table 1) and allowed us to map the residues that were largely responsible for this effect to the region encompassing *S. pomb*e residues _140_LAEGL_145_ (see Figure 6A). Thus, we conclude that the C-terminal portion of the fission yeast YG box contains determinants that limit formation of higher-order oligomers.

Inspection of the *S. pombe* YG box shows that it is a sequence outlier, even when compared to other fungal SMN orthologs. Indeed, the fungal consensus in this region is _140_LYEGQ_145_ (numbering per spSMN, see Supplementary Figure S3B). Although substitutions are clearly allowed, *S. pombe* is the only species among the nine clades we surveyed that have an alanine at position 141 (see Supplementary Figure S3B). To better model the vertebrate consensus, we generated an spL140Y,A141Y mutant construct and tested it *in vivo* for complementation in an *smn1* deletion background, as described above. As shown in Figure 3B, this allele fully rescued the growth defect of the null allele. When assayed *in vitro* for oligomerization using SEC-MALS, we found that it displayed a tetramer-octamer distribution similar to those of the human, fruitfly and nematode SMN•G2 complexes (Figure 6B). Furthermore, we note that in flies, the dmL207Y mutation (spL140Y) also improved the viability phenotype as compared to the WT Flag-*Smn* rescue transgene (Supplementary Figure S4), whereas expression of an SMA-causing mutation at the adjacent residue, hsY277C/dmY208C, significantly impaired viability (Figure 5A). Collectively, these findings implicate spAla141 as an anti-oligomerization determinant.

We therefore generated SMN•G2 constructs bearing the corresponding mutations in *Drosophila* and human SMN and determined their oligomerization status using SEC-MALS. As shown in Figure 6B and Table 1, the dmY208A and hsY277A complexes are consistent with a dimer-tetramer equilibrium. We generated a hs^1–275^-GCN4(IL) chimeric fusion as a control for a dimeric complex. As expected, this construct with Gemin2 forms obligate dimers (Table 1). Thus, when evolutionarily variant residues are considered, the SMN•G2 oligomerization results are completely concordant across multiple eukaryotic phyla, including *Chordata*, *Arthropoda*, *Nematoda* and *Ascomycota* (Table 1).

Having established the importance of *Drosophila* Tyr208 to the formation of higher-order SMN oligomers *in vitro*, we generated transgenic flies bearing a dmY208A mutation to study its effects *in vivo*. We assayed organismal viability, lethal stage and larval locomotion phenotypes in comparison to the previously described SMA missense mutation strain, dmY208C (44), as well as to wild-type controls. As shown in Figure 6C, control animals complete development and eclose at high frequency, unlike the two missense mutants. However, dmY208A animals display a significantly more severe phenotype than do the dmY208C mutants, as a small fraction of the latter reach adulthood. By contrast, none of the dmY208A mutants eclose as adults, and they are also significantly impaired during pupariation as compared to either the dmY208C or the WT control line (Figure 6C). Both missense mutations display SMA-like phenotypes early in development, appearing during larval stages. Locomotion analysis shows that larval crawling velocity is significantly reduced for both dmY208C and dmY208A (Figure 6D). Finally, intragenic complementation analysis of SMA-causing mutations within the YG box versus the Tudor domain suggest that these two domains perform independent functions (Supplementary Figure S5 and (21)).

On the basis of the experiments described above, we conclude that animals expressing YG box mutations that allow dimerization but prevent assembly of higher-order SMN oligomers are incompatible with metazoan viability. The results also call into question whether unicellular organisms like fission yeast require formation of SMN•G2 oligomers larger than tetramers to support cell growth.

### Defining the SMN oligomeric interface

The combined data from three different model systems highlight the importance of four distinct residues in the assembly of higher-order SMN multimers. Obviously, hsY277/dmY208/spA141 is one of those residues, as detailed above (Figures 5 and 6). In addition, we showed that mutations at hsH273/dmY204/spY137 shift the oligomeric equilibrium towards lower-order species (Figures 3–5). The other two residues are hsS266/dmA193/spS130 and hsS270/dmS201/spA134, as mutations at these positions are inviable *in vivo* and prevent formation of tetramers *in vitro* (Figure 3).

Among the lesser studied residues within the SMN YG box are those that comprise the S (serine)-motif (25), which for reasons that will become apparent, we have renamed as the s (small)-motif, Figure 1A. Due to the presence of conserved serine/threonine residues, especially within higher eukaryotic organisms, we and others initially assumed that phosphoregulation of these residues was somehow important for SMN biology. Indeed, mutation of hsS270/dmS201 to either aspartate or alanine has dramatic (and opposite) effects on SMNΔ7 protein stability in both human and *Drosophila* cells (52,53). Notably, this residue forms a key part of an E3 ubiquitin ligase phosphodegron (52), but the serine itself is not essential for viability in the context of full-length SMN, as dmS201A mutants are viable and fertile (Figure 5A). By contrast, substitution of a bulky Phe residue at this position renders the dmS201F protein unstable *in vivo* (32,54), and the animals are completely inviable (Figure 5A; (55)).

**Figure 7.**
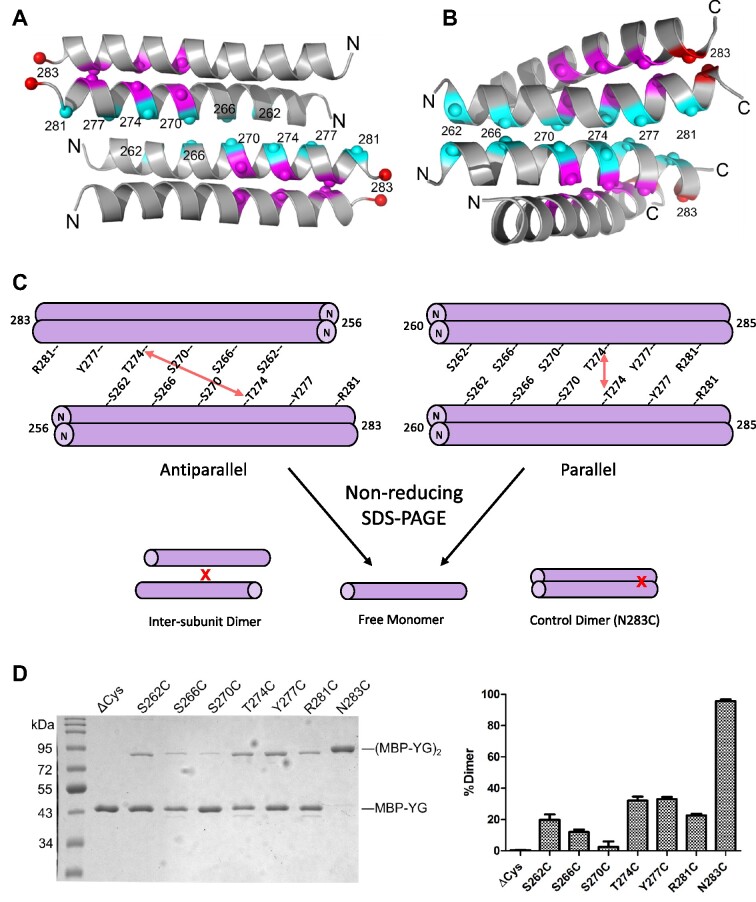
Disulfide crosslinking analysis and models of higher order SMN oligomerization. (**A**) Model of an antiparallel SMN YG box tetramer based on crystal contacts in a spSMN crystal structure (PDB ID: 7BB3). Numbering as per human SMN. (**B**) Model of a parallel YG box tetramer, based on s-motif helix–helix interactions observed in membrane proteins (see Materials and Methods and text for details). (**C**) Cartoon of disulfide crosslinking between YG box dimers containing single cysteine substitutions at indicated residues. Crosslinking between Thr274 residues is illustrated with a red arrow as an example. Disulfide crosslinking allows for resolution of dimers upon non-reducing SDS-PAGE. The N283C substitution serves as a positive control because hsN283 (marked in red in panels A and B) is within the dimer interface and is efficiently crosslinked. (**D**) SDS-PAGE of MBP-hsSMN YG box fusions treated with 160 μM diamide for 60 min (left). The graph on the right shows the average fraction of dimers formed from three independent crosslinking experiments. Error bars show standard deviation. Note that MBP-YG box fusions treated with 1 mM DTT rather than diamide are monomeric (see Supplementary Figure S8).

Glutamine scanning mutagenesis of various small residues in the yeast YG box (Figure 3A) reveals that insertion of a bulky and polar Gln residue can be tolerated at certain positions in spSMN (e.g. spA145Q and spA141Q), whereas at other positions (e.g. spS130Q and spA134Q) it cannot. Quizzically, the two s-motif residues that are intolerant to Gln substitution are located at positions that do not lie along the direct YG zipper dimerization interface (Figure 1). To better visualize this arrangement, we highlighted the location of the s-motif residues on the surface of the hsSMN YG box dimer. As illustrated in Figure 7A, B, the Gly residues directly involved in SMN dimer formation are located along one face of the alpha helix, whereas the residues implicated in higher-order multimerization are on the opposite side. The alignment of conserved small residues (Ser266, Ser270 and Thr274) along the same face of the helix suggests the existence of a novel binding interface that could be used to generate higher order oligomers.

### Parallel versus antiparallel SMN tetramers

Tetramers can form by two distinct mechanisms: one in which all four subunits are equivalent (symmetric bundle), and one that involves self-association of dimeric subunits (dimer of dimers). In a symmetrically bundled tetramer, the same residues are involved in forming the inter-subunit contacts of both the dimeric and tetrameric forms, whereas in the dimer of dimers model, different binding surfaces are used for dimerization versus tetramerization. Previously, we showed that spSMN forms tetramers *in vitro* via the association of stable dimers (24). We therefore considered whether SMN•G2 dimers might associate in a parallel vs. antiparallel fashion.

We used SEC-SAXS data from spSMN•G2 constructs to evaluate the likelihood of parallel versus antiparallel arrangements of YG boxes in an SMN tetramer. To obtain data for the spSMN•G2 dimer, we determined the radius of gyration (*R*_g_) and maximum dimension (*D*_max_) for spSMN(GCN4IL)•G2, which forms obligate dimers (Supplementary Table S4). We previously reported *R*_g_ and *D*_max_ values for tetrameric spSMN•G2 (24), also listed in Supplementary Table S4. A comparison of SAXS results for the two complexes leads to the surprising conclusion that tetrameric SMN is only slightly larger in size (*D*_max_ = 276Å) than dimeric SMN (*D*_max_ = 233Å). As illustrated in Supplementary Figure S7, the observed spatial restriction of the tetramer can be more readily explained by a parallel arrangement of dimers. In an independent method of analysis, the *R*_g_ values of the dimer and tetramer can be used to calculate the distance (*L*_1_) between centers of mass of the dimers within the tetrameric complex by application of the parallel axis theorem (56). For the wild-type spSMN•G2 tetramer, this calculated value is *L*_1_ = 106 Å, a distance that could be consistent with either a parallel or antiparallel arrangement of dimers (Supplementary Figure S7). Thus, SAXS analysis leads to a preference for parallel tetramers based on maximum distance values, but not as measured by radii of gyration.

While this manuscript was under consideration (57), the crystal structure of a non-native fusion between the Gem2 domain and YG box of spSMN was reported (58). In that structure (PDB ID 7BB3), the YG box dimers interact to form a polymer of antiparallel stacked dimers in the crystal lattice, with the Gem2 domain helices forming an independent array of hydrophobic interactions. As anticipated, the s-motif residues play key roles in the observed crystal lattice contacts, and provide a plausible molecular model for an antiparallel YG box tetramer. However, other YG box residues highlighted in our study of native metazoan SMN complexes (e.g. hsY277 and hsH273) play correspondingly less prominent roles in the reported yeast fusion structure. We therefore carried out disulfide crosslinking experiments to directly test parallel vs. antiparallel models of YG box oligomers.

MBP-YG box fusion constructs of yeast and human SMN were engineered to contain a single cysteine residue at key positions. Because there are no cysteine residues within MBP-hsSMN^252-294^(C289A), we used this construct (ΔCys) as the starting point for the human YG box. These fusions have been well characterized (25) and, as described above, recapitulate the oligomeric properties of native SMN•G2 complexes. Moreover, the crystal structure of the yeast MBP-YG box dimer (24) is entirely congruent with that of the dimeric subunits reported by Fischer and colleagues (58). An outline of the experiment is illustrated in Figure 7C. As shown in Figure 7D, the ΔCys negative control does not form disulfide linkages, whereas the hsSMN^N283C^ (N283C) positive control is efficiently crosslinked, with nearly complete conversion to dimers on SDS-PAGE. Note that hsSMN Asn283 is located within the dimeric interface (Figure 7A) and that hsSMN^N283C^ corresponds to spSMN^S147C^, which we previously used as a positive control for crosslinking yeast YG box dimers (24).

We tested the crosslinking efficiencies of six cysteine substitutions at residues predicted to form the oligomeric interface (Figure 7D). S266C and S270C are the least efficiently crosslinked substitutions, indicating that these s-motif residues are not well-positioned for self-interaction along the dimer–dimer interface. T274C and Y277C are crosslinked at the highest levels among the six residues tested, indicating that they are located close to their partners in the tetramer interface. The remaining mutants, S262C and R281C, displayed intermediate crosslinking efficiencies (Figure 7D). Hence, the human crosslinking data are more consistent with a parallel interface between YG box dimers. We also carried out crosslinking experiments on the corresponding yeast MBP-YG box fusions. Those experiments also showed poor crosslinking efficiency for the critical s-motif residues S130C and A134C and better supported a parallel model (Supplementary Figure S8). The primary difference compared to the hsSMN results was a lack of strong crosslinking activity for spA141C, which corresponds to hsY277C (see Discussion). Altogether, these data are most consistent with the hypothesis that SMN YG box dimers associate in a parallel fashion to form tetramers.

### A structural model for SMN oligomer formation

Models of human YG box tetramers are shown in Figure 7A, B. For the antiparallel model, we used the crystal packing observed in the structure of the spSMN fusion construct mentioned above (58), to define the interface. In this case, the tetramer is formed by a left-handed crossing of helices (Figure 7A). To generate a plausible model for a parallel YG box tetramer, we searched for examples of helix-helix interactions involving s-motif residues (Figure 1A, Supplementary Figure S1A) such as serine, alanine, and threonine. Helix-helix packing that features small residues at the interface is common among membrane proteins, where the interfaces are enriched for Ala, Ser, Thr and Gly residues, and the helix crossing is generally right-handed (59). Indeed, an extreme example of this type of helix-helix interaction is the glycine zipper, where Gly residues mediate intimate contact between the helical backbones as occurs in the SMN YG box dimer. In contrast, the well-studied coiled-coil interface is enriched in Leu, Ile, and Val residues and the crossing is left-handed (60,61).

We therefore generated a parallel YG box tetramer model based on a previously observed small residue interface, using the glycerol facilitator protein structure (62) as a template. Superimposing one helix from each of two YG box dimers onto a helical dimer from PDB entry 1FX8 (62), the resulting model places the conserved s-motif residues, along with His273 and Tyr277, at or near the interface between SMN dimers. Note that a left-handed crossing of dimers within a parallel SMN tetramer is also possible, but not modeled here. In summary, the models shown in Figure 7A, B illustrate both antiparallel and parallel docking of YG box dimers that utilize both left- and right-handed crossing of the YG box helices.

## DISCUSSION

Transmembrane proteins frequently contain GxxxG motifs that promote dimerization (61). An extension of this motif (G,A,S)xxxGxxx(G,S,T) is known as a glycine zipper (35), which is thought to mediate oligomerization. Although helices bearing these motifs interact with right-handed crossing angles, glycine zipper proteins form symmetrically bundled oligomers that associate in a front-to-back fashion, whereas GxxxG dimers employ face-to-face packing (35,63). The SMN YG box contains an extended GxxxGxxxG motif, however the Gly residues are face-to-face as in GxxxG dimers (Figure 1B). This unique feature suggests that once a YG zipper dimer forms, that same binding surface should no longer be available to generate oligomers. Our experimental data are not only consistent with this model, but they identify specific YG box residues that mediate formation of higher-order multimers.

### Structural aspects of SMN oligomerization

Residues within the tetrameric SMN interface (Figure 7A,B) are not normally found at interfacial positions of soluble protein coiled-coils, which are dominated by Leu, Ile, and Val (60). In SMN, the core interfaces involved in forming both dimers and oligomers employ small residues like Ser, Ala, Thr and Gly. In the parallel and antiparallel models, Ser270 is buried in the tetrameric interface where inter-helical hydrogen bonds could be formed with the polypeptide backbone, as observed for membrane proteins (59). Such positioning would explain the sensitivity of the corresponding spSMN and dmSMN residues to substitution by larger side chains, but not by smaller ones (Figures 3 and 5). Both models also explain how formation of higher-order multimers could protect SMN from degradation via sequestration of the Ser270 phosphodegron (52), which is solvent exposed in the dimer.

Two other highly conserved s-motif residues, Ser266 and Thr274, are located in the tetrameric interfaces of both models. A role for Ser266 in the antiparallel model is unclear, but Thr274 makes a hydrogen bond to Trp267 that is one of the few specific interactions in the interface. The SMA mutation T274I is expected to disrupt the antiparallel interface shown in Figure 7A due to a clash with Trp267 and indeed, we found that spSMN T138I is deficient in oligomerization (Figure 3). However, hsSMN T274I is only moderately affected, arguing against this close contact in human SMN oligomers. hsT274C was one of the two cysteine mutants most efficiently crosslinked (Figure 7D), yet the corresponding residues are far apart in the antiparallel model (Figure 7A,C). In contrast, Ser270 is expected to be close to its partner in the antiparallel model, yet the S270C mutant is least efficiently crosslinked (Figure 7A,D).

A key finding of this study is identification of hsY277/dmY208/spA141 as a major determinant of higher-order oligomerization. A large hydrophobic residue is strongly conserved at Tyr277, whereas the Ala found in *S. pombe* is an outlier. The reduction in the extent of spSMN oligomerization, compared to that of its metazoan orthologs (Table 1), can be attributed to the lack of bulky hydrophobic residues at Leu140 and Ala141, as Tyr substitution of these residues promotes higher-order oligomerization (Figure 6B). The same argument explains the loss of higher-order multimers we observed for the hsY277A and dmY208A mutants (Figure 6, Table 1). Indeed, the strong temperature dependence of SMN oligomerization we observed for the metazoan complexes (Figure 2) also suggests a hydrophobic driving force. The defective phenotypes of hsY277C and dmY208C are consistent with the idea that this residue plays a key role in formation of higher-order multimers and that oligomers larger than SMN dimers are required for proper animal development. A critical test for any structural model would therefore be to explain these phenotypes. The antiparallel model shown in Figure 7A is derived from spSMN, where the residue corresponding to hsY277 is alanine. Residues near the N-terminus of the YG box that are likely to interact with a tyrosine substituted at this position are poorly conserved, making it difficult to rationalize how this residue mediates formation of antiparallel oligomers. In support of a parallel arrangement, we note that an A141Y substitution increased the disulfide crosslinking efficiency of an A145C mutant (Supplementary Figure S8), as would be expected for a mutation that promotes oligomerization (Figure 6A, B).

Another key residue is hsH273/dmY204/spY137. Evolutionarily speaking, His273 is usually a Tyr in the SMN proteins of lower organisms, with Gln present in a very small number of cases (Figure 1A, Supplementary Figure S1A). There is a strong correlation between the presence of His at position 273 (hsSMN numbering) and a large hydrophobic at 280 (see Supplementary Figures S1A and S3). When Tyr is present at position 273, the residue at 280 is more variable and is often not hydrophobic. This finding suggests that His273 might be less effective than Tyr in this position but is compensated by a strong hydrophobic residue at position 280, and most often a flanking hydrophobic at 278. These observations provide a plausible explanation for the lethal phenotype and reduced oligomerization of the Y204H substitution in dmSMN, where the residues corresponding to Met278 and Phe280 in hsSMN are Gln and Lys, respectively (Table 1, Figure 5). Insertion of sequences that place Met and Leu in these positions (+MGLR) results in a more ‘histidine friendly’ environment and a viable phenotype (Figure 5). The antiparallel tetramer model (Figure 7D) provides no insight into these observations, because a hydrophobic residue at 280 (spLeu144) is predicted to be juxtaposed with an acidic Asp- and Glu-rich region located in the N-terminal region of metazoan SMN orthologs (Supplementary Figure S1A).

Additional residues contribute to the interfaces shown in Figure 7A, B. Leu260, Met263 and Trp267 form conserved hydrophobic interactions in the antiparallel model, with Trp267 playing a particularly important role at the center of the interface. Met269, Thr274, Tyr276, Met278 and Phe280 would be expected to provide flanking hydrophobic interactions in both models. In summary, an antiparallel interface between YG box dimers explains some of the genetic and biochemical observations reported here but is inconsistent with others. Moreover, it is not supported by disulfide crosslinking experiments. In contrast, a parallel model is consistent with the crosslinking results and could in principle explain the available genetic and biochemical data, but no high-resolution structure is yet available for comparison. Because many models of both parallel and antiparallel dimers-of-dimers are possible (e.g. differing by helical crossing angles and points of interaction), further structural investigations are essential. It will be particularly important to obtain structural data for YG box oligomers in the context of native SMN complexes.

### SMN multimerization and biomolecular condensation

Protein oligomerization is thought to underlie a phenomenon that allows homogenous solutions of macromolecules to separate, or ‘demix,’ into two co-existing liquid phases (64). This biomolecular condensation makes it possible to create membraneless organelles or compartments with elevated protein concentrations that serve to accelerate biochemical reactions or to sequester key factors away from the cellular milieu (65,66). As with most things in nature, the positive benefits provided by multimerization come with a down-side: oligomeric proteins also have the potential to form dysfunctional or pathogenic aggregates (67–70). Molecular mechanisms underlying a wide variety of physiological and pathological processes are thus being re-examined through the lens of liquid-liquid phase separation (71). The models we outline in Figure 7 explain a large body of genetic, phylogenetic and biochemical data. Because the SMN•G2 tetramer has two binding surfaces available for self-interaction, the models provide an obvious mechanism for forming large polymers.

What factors limit the extent of SMN oligomerization? *In vitro*, metazoan SMN•G2 complexes in the μM range exist primarily in a tetramer-octamer equilibrium, and their hydrodynamic properties show that they adopt highly extended (non-globular) conformations. Clearly, the N-terminal portion of SMN is an important determinant of its overall solubility and oligomerization potential, as YG box constructs tagged with small epitopes or synthetic YG box peptides form large, insoluble aggregates. By analyzing a series of MBP-YG box fusions, we found that we could control the size of the complexes formed (*n* = 1, 2, 4 or 8) by very small changes in the length of the linker [(25) and this work]. The amino terminal domains of SMN, along with Gemin2, plausibly limit multimerization. Thus, steric factors including N-terminal composition and linker length are critical determinants in regulating the extent of YG box oligomerization.

*In vivo*, post-translational modifications (PTMs) and the presence of additional binding partners are almost certain to play important roles in regulating SMN oligomerization. Gemin2 and the Sm proteins are known to bind directly to the Gem2 and Tudor domains of SMN, respectively (reviewed in (4)). In addition, two other Gemin subcomplexes associate with the YG box. The Gemin3-4-5 and Gemin6-7-8 subunits are tethered to SMN via Gemin3 and Gemin8, respectively (19). Binding of these additional subunits to the oligomeric core of SMN•G2 will doubtless have an effect, but it is hard to predict its direction. Should these PTMs and binding interactions work to increase the local concentration of SMN, they would be predicted to drive demixing of the complex into phase-separated membraneless organelles (72) such as nuclear Cajal and Gemini bodies, or cytoplasmic stress granules and U bodies. Such a finding would also suggest that SMN might carry out different functions in different cellular locales, and that these functions might depend on its oligomerization status. Thus, an important goal for the future is to elucidate the nature of the interactions that give rise to the underlying oligomeric heterogeneity of SMN subcomplexes *in vivo*. The work here not only demonstrates that formation of higher-order SMN complexes is required for metazoan viability, but it also provides key mechanistic insight into their assembly.

## DATA AVAILABILITY

SAXS data generated as part of this work have been deposited at the SASDB under accession codes: SASDL35, SASDL45, SASDL55, SASDL65, SASDL75, SASDL85 and SASDL95.

## Supplementary Material

gkab508_Supplemental_Files

## Notes

Impact Statement: Survival Motor Neuron protein multimerization is required for metazoan life and depends on specific YG box domain residues that are not involved in dimer formation.
