# Supplementary material for: Assembly of higher-order SMN oligomers is essential for metazoan viability and requires an exposed structural motif present in the YG zipper dimer
Source: Nucleic Acids Res. 2021 Jun 28;49(13):7644–64. doi: 10.1093/nar/gkab508 (PMC8287954; doi:10.1093/nar/gkab508)
Supplement: gkab508_Supplemental_Files [file gkab508_supplemental_files.zip › Gupta NAR suppl tables.docx]

**Supplementary Tables**

**Table S1. Oligomeric Properties of SMN•G2 Chimeras.**

| SMN•G2 Construct | SEC-MALS M_w_ (20°C)^B^ | Peak Concentration (µM)^C^ | Oligomer State |
| --- | --- | --- | --- |
| *H.sapiens* | ~247 kD (200-600) | 0.14 | Tetramer-Octamer^A^ |
| *H.sapiens* ∆5 | ~231 kD (188-454) | 0.7 | Tetramer-Octamer |
| SMN∆7 | ~66 kD (58-74) | 0.2 | Monomer^A^ |
| *S.pombe* | ~150 kD (110-180) | 2.3 | Dimer-Tetramer^A^ |
| *C.elegans* | ~326 kD (250-450) | 0.2 | Tetramer-Octamer |
| *D.melanogaster* | ~345 kD (202-380) | 0.4 | Tetramer-Octamer |
| Hs^1-275^-Sp^140-152^ | ~128 kD (88-227) | 2.3 | Dimer |
| Hs^1-279^-Sp^144-152^ | ~285 kD (119-640) | 0.1 | Tetramer-Octamer |
| Hs^1-275^-GCN4(IL) | ~113 kD (80-113) | 0.3 | Dimer |
| Hs^1-251^-GCN4(LI) | ~190 kD (190-400) | 0.0 | Tetramer-Octamer |
| Sp^1-117^-GCN4(IL) | ~85 kD (79-85) | 2.1 | Dimer |
| Sp^1-117^-GCN4(IL)-spSMN^145-152^ | *n.d.* | *n.d.* | Dimer^A^ |
| Sp^1-117^-GCN4(LI) | ~125 kD (90-128) | 1.1 | Dimer-Trimer |
| Sp^1-117^-GCN4(II) | ~113 kD (73-120) | 0.7 | Dimer-Trimer |
| Sp^1-141^-Ce^187-197^ | ~160 kD (120-170) | 0.5 | Dimer-Tetramer |
| Ce^2-184^-Sp^140-152^ | ~137 kD (137 – 610) | 0.9 | Dimer |

^A^See ref. (24)

^B^Shown is the MALS molecular weight determined at UV peak. In parentheses, the range of molecular masses observed from UV peak half-height to half-height is shown.

^C^As determined by refractive index.

**Table S2. Biophysical Properties of *sp*SMN•Gemin2 Mutants (related to Figure 3)**

M = Monomer, D = Dimer, T = Tetramer, O = Octamer, SS = Single Species

|  | Sedimentation Equilibrium Analytical Ultracentrifugation^A^ | | | | | SEC-MALS^C^ |
| --- | --- | --- | --- | --- | --- | --- |
| Construct | Speeds (krpm) | Conc. (µM) | Model of Association | K_d (_µM) | χ^2^ | Mass Profile |
| Wild-type^B^ | 8,10,12,14 | 2.2, 3.3, 4.3 | 2-4 | 1.0 ± 0.9 |  | D-T |
| Wild-type | 8,10,12,14 | 2.3 | 2-4 | 2.8 ± 0.4 | 0.9 | D-T |
| S130Q | 12,14,16 | 5.6, 3.6 | 2-4 | 4.0 ± 0.25 | 1.4 | D>T |
| A134Q | 12,14,16 | 9.2, 7.9 | 2-4 | 14.3 ± 0.09 | 0.6 | D |
| Y136C | 8,10,12,14 | 6.5 | SS 1:1 | *n.a.* | 0.9 | M |
| Y137R | 8,10,12,14 | 2.2 | 2-4 | 274 ± 32 | 1.0 | D |
| T138I | *n.d.* | *n.d.* | *n.d.* | *n.d.* | *n.d.* | M+ |
| G139S | 8,10,12,14 | 1.2 | 1-2 | 4.5 ± 0.75 | 0.4 | M-D |
| L140Y,A141Y | *n.d.* | *n.d.* | *n.d.* | *n.d.* | *n.d.* | T-O |
| A141Q | 12,14,16 | 6.3, 4.6 | 2-4 | 0.55 ± 0.07 | 1.7 | D-T |
| E142R | *n.d.* | *n.d.* | *n.d.* | *n.d.* | *n.d.* | D-T |
| G143V | *n.d.* | *n.d.* | *n.d.* | *n.d.* | *n.d.* | D-T |
| A145Q | 12,14,16 | 3.5 | 2-4 | 1.1 ± 0.3 | 0.9 | D-T |
| spSMN^1-141^-ceSMN^187-end^•Gemin2 | *n.d.* | *n.d.* | *n.d.* | *n.d.* | *n.d.* | D-T |
| spSMN(GCN4IL)•Gemin2 | 8,10,12,14 | 1.4 | SS 2:2 | *n.a.* | 2.1 | D |

*n.d.* = not determined

*n.a.* = not applicable

^A.^Analyses were performed in 20 mM Tris 7.4, 200 mM NaCl, 5 mM DTT and data analyzed using the program SEDPHAT (74).

^B.^in 20 mM Na/KPO_4_ pH 7.0, 150 mM NaCl, 1 mM DTT (24).

^C.^ Analyses were performed in 20 mM Tris 7.4, 200 mM NaCl, 5 mM DTT with a Superdex 200 10/300 column at room temperature.

**Table S3. Parameters derived from Size-Exclusion Chromatography In-line with Small-Angle X-ray Scattering (SEC-SAXS)**

| Sample^1^ | Guinier | | GNOM | | ^2^P_x_ | Mass (kD) | | Oligomer |
| --- | --- | --- | --- | --- | --- | --- | --- | --- |
|  | qR_g_ | R_g_ (Å) | R_g_ (Å) | D_max_ (Å) |  | ^3^Q_r_ | ^4^Porod |  |
| dmSMN•Gemin2 | 0.71 – 1.85 | 82.0 ± 0.8 | 83.7 ± 0.9 | 271 | 2.7 | 472 | 390 | Octamer |
| MBP-dmSMN^189-220^ | 0.54 – 1.14 | 56.1 ± 0.7 | 53.0 ± 0.2 | 179 | 4.0 | 345 | 409 | Octamer |
|  | 0.56 – 1.21 | 38.0 ± 0.4 | 41.2 ± 0.1 | 113 | 4.0 | 239 | 227 | Tetramer |

^1^In 20 mM Na/KPO_4_ pH 7.0, 300 mM NaCl, 1 mM DTT

^2^Porod exponent. Values near ~4 indicate compactness, whereas lower values between <2-3 indicate significant lack of compactness and increased volumes (75). These values were determined using the program ScÅtter (https://bl1231.als.lbl.gov/scatter/).

^3^Mass determinations (MM) using the Q_r_ invariant (76) were determined using the program RAW(77).

^4^Porod Volume (V_p_). This figure can be used to estimate the mass of compact proteins, where V_p_/1.6 ~ MM.

**Table S4. Parameters derived from Small-Angle X-ray Scattering (SAXS) for spSMN(GCN4IL)•Gemin2**

| Conc.^1^ | Guinier | | GNOM | | ^2^P_x_ | Mass (kD) | | Oligomer |
| --- | --- | --- | --- | --- | --- | --- | --- | --- |
| (mg/mL) | qR_g_ | R_g_ (Å) | R_g_ (Å) | D_max_ (Å) |  | ^3^Q_r_ | ^4^Porod |  |
| 12 | 0.52 – 1.10 | 65.1 ± 0.4 | 67.5 ± 0.3 | 233 | 3.5 | 113 | 126 | Dimer |
| 6 | 0.52 – 1.10 | 65.3 ± 0.7 | 68.7 ± 0.3 | 229 | 3.7 | 115 | 133 | Dimer |
| 4 | 0.57 – 1.15 | 63.9 ± 0.8 | 66.8 ± 0.3 | 219 | 3.6 | 110 | 127 | Dimer |
| 3 | 0.57 – 1.15 | 63.8 ± 1.1 | 65.7 ± 0.4 | 199 | 3.9 | 106 | 121 | Dimer |

^1^In 20 mM Na/KPO_4_ pH 7.0, 150 mM NaCl, 1 mM DTT

^2^Porod exponent. Values near ~4 indicate compactness, whereas lower values between <2-3 indicate significant lack of compactness and increased volumes (75). These values were determined using the program ScÅtter (https://bl1231.als.lbl.gov/scatter/).

^3^Mass determinations (MM) using the Q_r_ invariant (76) were determined using the program RAW(77).

^4^Porod Volume (V_p_). This figure can be used to estimate the mass of compact proteins, where V_p_/1.6 ~ MM.
