# Supplementary material for: Assembly of higher-order SMN oligomers is essential for metazoan viability and requires an exposed structural motif present in the YG zipper dimer
Source: Nucleic Acids Res. 2021 Jun 28;49(13):7644–64. doi: 10.1093/nar/gkab508 (PMC8287954; doi:10.1093/nar/gkab508)
Supplement: gkab508_Supplemental_Files [file gkab508_supplemental_files.zip › Gupta NAR suppl2.pdf]

Supplemental Materials

Assembly of higher-order SMN oligomers is essential for metazoan viability and requires an exposed structural motif present in the YG zipper dimer

Kushol Gupta[1], Ying Wen[2], Nisha S. Ninan[1], Amanda C. Raimer[2,3], Robert Sharp[1], Ashlyn M. Spring[2,4], Kathryn L. Sarachan[1], Meghan C. Johnson[4], Gregory D. Van Duyne*[1] and A. Gregory Matera*[2,3,4]

[1]Department of Biochemistry & Biophysics, Perelman School of Medicine, University of Pennsylvania, Philadelphia, PA 19105-6059, USA.
[2]Integrative Program for Biological & Genome Sciences, Lineberger Comprehensive Cancer Center, University of North Carolina, Chapel Hill, NC 27599, USA.
[3]Curriculum in Genetics and Molecular Biology, Department of Genetics, University of North Carolina, Chapel Hill, NC 27599, USA.
[4]Department of Biology, University of North Carolina, Chapel Hill, NC 27599, USA.

*Address correspondence to:

Gregory D. Van Duyne, Department of Biochemistry & Biophysics, Perelman School of Medicine at the University of Pennsylvania, 809C Stellar-Chance Building, 422 Curie Boulevard, Philadelphia, PA 19104-6059 (Tel) 215-898-3058. Email:vanduyne@pennmedicine.upenn.edu

A. Gregory Matera, Integrative Program for Biological & Genome Sciences, University of North Carolina, 3352 Genome Sciences Building CB#7100, Chapel Hill, NC 27599. (Tel) 919-962-4567. Email:matera@unc.edu

YG Box: Extended Phylogenetic Comparison

	——— YG Box ———
Human	DDADALGSMLISWYMSGYHTGYYMGFRQNQKEGRCSHSLN
Dog	DDADALGSMLISWYMSGYHTGYYMGFKQNQKEGRCSHFN
Pig	DDADALGSMLISWYMSGYHTGYYMGFRQNQKEGRCSHFN
Mouse	DDTDALGSMLISWYMSGYHTGYYMGFRQNKKEGKCSHTN
Chicken	EDDEALGSMLIAWYMSGYHTGYYLGLKQSRMEAALEREAYLK
Frog	EDDEALGSMLISWYMSGYHTGYYLGLKQGRMESSIGKPPHQK
Gator	EDDEALGSMLIAWYMSGYHTGYYLGLKQSRMEAALDRHPDPK
Snake	DDDEALGSMLIAWYMSGYHTGYYLGLKQGRMEATLERHAHSK
Gekko	EDDEALGSMLIAWYMSGYHTGYYLGLKQSRMEATSGRDAHSK
Killifish	VDDEALGSVLISWYMSGYHTGYYLGLKQGRKEANKWTKLHHK
Zebrafish	EDDEALGSMLISWYMSGYHTGYYMGLRQGRKEAAASKKSHRK
Fugu	VDDEALGSMLISWYMSGYHTGFYLGLKEGRKKASNWTKPHHR
Arowana	SDVAELSSMLLSWYLCGYHTGYYMALQQTNSSHEKTKKKYK[10aa]
Catfish	EDDEALGSMLISWYMSGYHTGYYLGLKQGRKEAAAKKSHYK
Shark	EDDEALGSMLIAWYMSGYHTGYYLGLKQGRAEEALGKSSHRK
Coelacanth	DADSTLVCMLLAWYMSGYHTGYYMGLKHGQAKATGSSQKKHPKRK
Octopus	DNNEALCSMLMSWYMSGYHTGYYQGLKSKQN
Urchin	MDKEALHSMLMSWYMSGYHTGYYEGMKKSKTSSHSATSKPK[42aa]
Oyster	GDNEAMCSMLMAWYMSGYHTGYYQGLKQGRQQGGTSPHPDSFR
Mollusk	GDNEAMCSMLMAWYMSGYHTGYYQGLKQGRQQGGTSPHPDSFR
Anemone	EDNEALASMLMSWYLSGYYTGYYQGTQRQHISHNDRTSQTT[23aa]
Coral	HDNDALASMLMAWYLSGYHTGYFQAMQNFRHESSMGSNEAQ[19aa]
Hydra	GDEEALAGMLMSWYMSGYHTGYYQGMHFLNRDSESKKIKNN[97aa]
Ciona	LSKDALSNMFASWYMAGYQTGFHRGLASSCCKNNCKK
Crab	TDDEALASMLMSWYMSGYHTGYYQALRQMRSESCECQGHIDKKCCLHCSS
Daphnia	MDSDSLYSMLMSWYMAGYHTGYYQGVQQRNKRKANSSGSS
Psyllid	DTTESLSAVLMAWYMAGYHTGRYEASLGLNRNKTFGRPQSQQVEKCCDHK
Bug	DESDALSAMLMAWYMSGYHTGYYQGLTRSTPSGSGIREGKSNTSRLNN
WaterTick	KESDALSSMLMAWYMSGYHTGYYQALQQSQQVEKCCDHK
Mite	EGDEALAAMLISWYISGYHTGYYTAVRNQSKG
Aphid	TEREALTSMLMSYYMSGFHTGYYLGIKQKNSEN
Ant	TDADALSSMLMSWYLSGFHTGYYHGLKQAKNQQKRRNC
Spider	SNDPSLSAMLVSWYMAGYYTGLHQVCI
Wasp	NDADALSSMLMSWYISGFHTGYYHGLKQAQSNQHRRKT
Honeybee	NDAEALSSMLMSWYISGFHTGYYHGLKQAEKNQTKRKNC
Mosquito	VESENLSAMLMSWYMSGYYTGLYHGQRMSQQQQHTQQKRARQS
Silkmoth	SEQQAISSMLLSWYMSGYYTGLYQGMKRSKENNKNV
Housefly	EDSEHLSAMLMSWYMSGYYTGVYQGMQMAKSKTKK
D.pseudo	GEEQDLMSMLTAWYMSGYYTGYFQGKKAIPRQVEKKKTPKK
D.melano	GAEQDFVAMLTAWYMSGYYTGLYQGKKEASTTSGKKKTPKK
Nematode	NQKEAMNSMLMSWYMSGYHTGYYQGLADQKNVQN
HookWrm	DEAEALSSMLMAWYMSGYHTGYYQAIRDMNSNS
FlatWrm	NDENAVRNLLNSWYMCGYQTGYLTALKKGARECV
TapeWrm	NDENAVRNLLNSWYMCGYQTGYLTALKKGTRECV
WhipWrm	KVDNALNAMLISWYMAGYHAGFYQAGTHSWFVMAYGFSVCSG[36aa]
AcornWrm	GDEDALFSMLISWYMSGYHTGYYQGLKASRGESLQNTPQRNE[24aa]
Priapulus	IDNDVLSSMLISWYMSGYHTGYYQGLKTAKLEALKKRKDDVP[27aa]
Lingula	GDNEALCSMLMSWYMSGYHTGYYQGLKDAKKQQHGHQPHKDSSDVKR
S.rosetta	AQEAALANMLMSWYQSGFYTGYYQALQQLQQQQQE
Dicty	QGDDELADLLLSWYYSGYYTGVYQERKRNSRLNQTPHPNHIN[69aa]
Neurosp	VQDEELKKLLMSWYYAGYYTGLYEGKQKALHEQAQQ
B.bassia	GRDDNLRKLLMSWYYAGYYTGLHEGQQQQTQAQQQPQ
S.pombe	TYDETYKKLIMSWYYAGYYTGLAEGLAKSEQRKD
P.italic	VQDESLKNLMMSWYYAGYYTGLHEGQQQTNSNQSS
Trypano	RLPADIRQLLVAYFNAGYEAGYYVGKRDGSSKVGGKRARGE
Cotton	SCETDLTVLLNAWYSAGFYTGKYLVEQSIAKRRQ
Corn	NLDSDLAALLNSWYAAGFYTCRYLMLQSTKNSRP
Legume	DSATDLTAVLNAWYSAGFHTGKYLAEQSIGNRRQI
Jute	TSETDLTVLLNAWYSAGFHTGKYLMEQSIAKKQHK
Consensus	LxSMLxSWYxSGYxTGYxxGL

Figure S1A. Extended phylogenetic alignment of the SMN YG Box. Phylogenetic analysis of SMN C-termini from a wide variety of eukaryotes. Conserved glycine residues are shaded in magenta, hydrophobic residues are in green, and polar residues in teal. Relates to Fig. 1A.

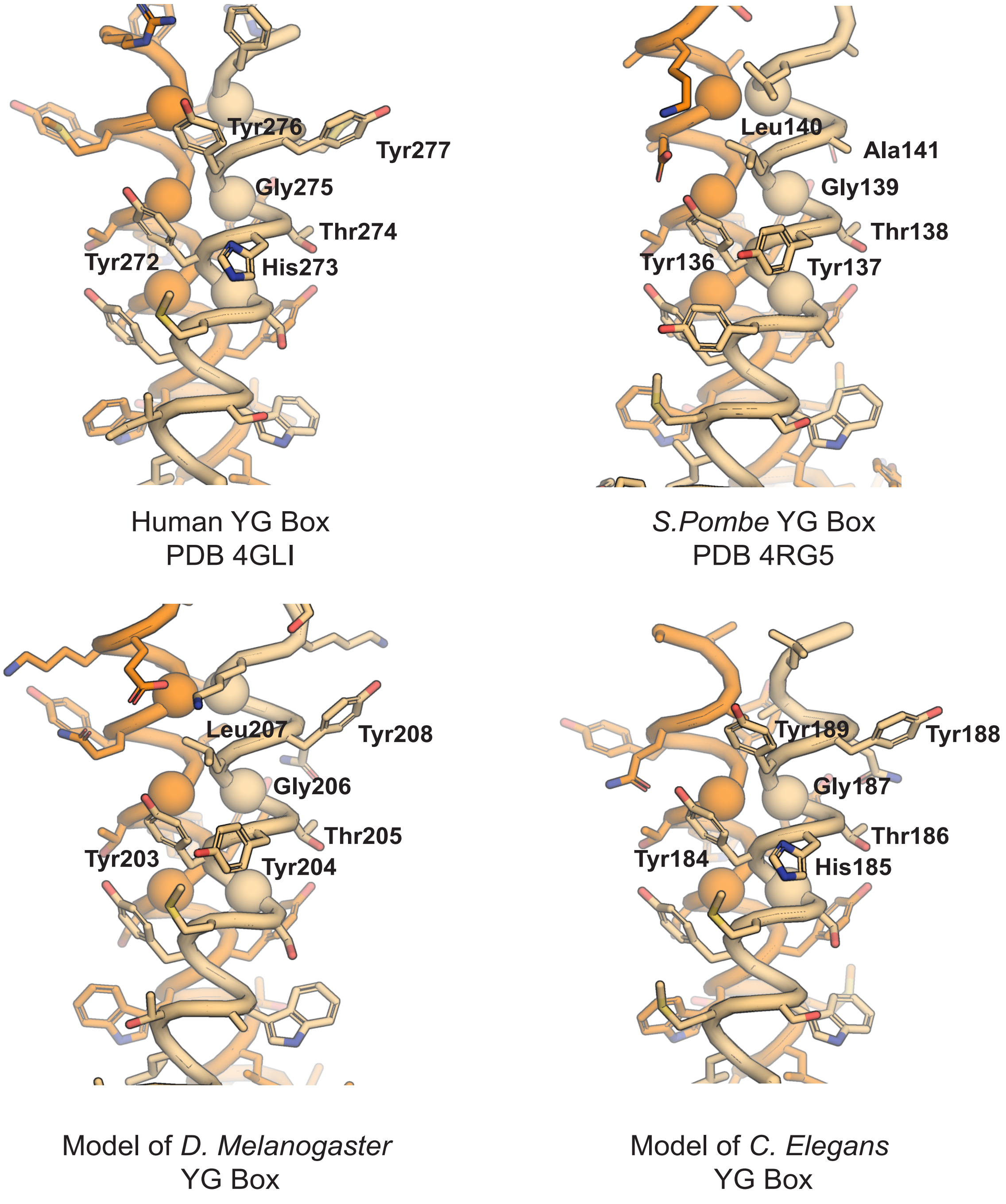

Figure S1B. Atomic views of the SMN YG-box dimer. Shown in the upper panels are the experimental atomic structures of the human (left, PDB 4GLI; Ref. 25) and yeast (right, PDB 4RG5 (24) YG boxes. In the lower panels are models of the fly (left) and nematode (right) derived from the 4GLI structure. Shown as spheres in each panel are well-conserved glycine residues at C_α. The residues corresponding to amino acids 272-277 in human are labelled in each panel. The figure was rendered using the program PYMOL (73). Relates to Fig. 1B

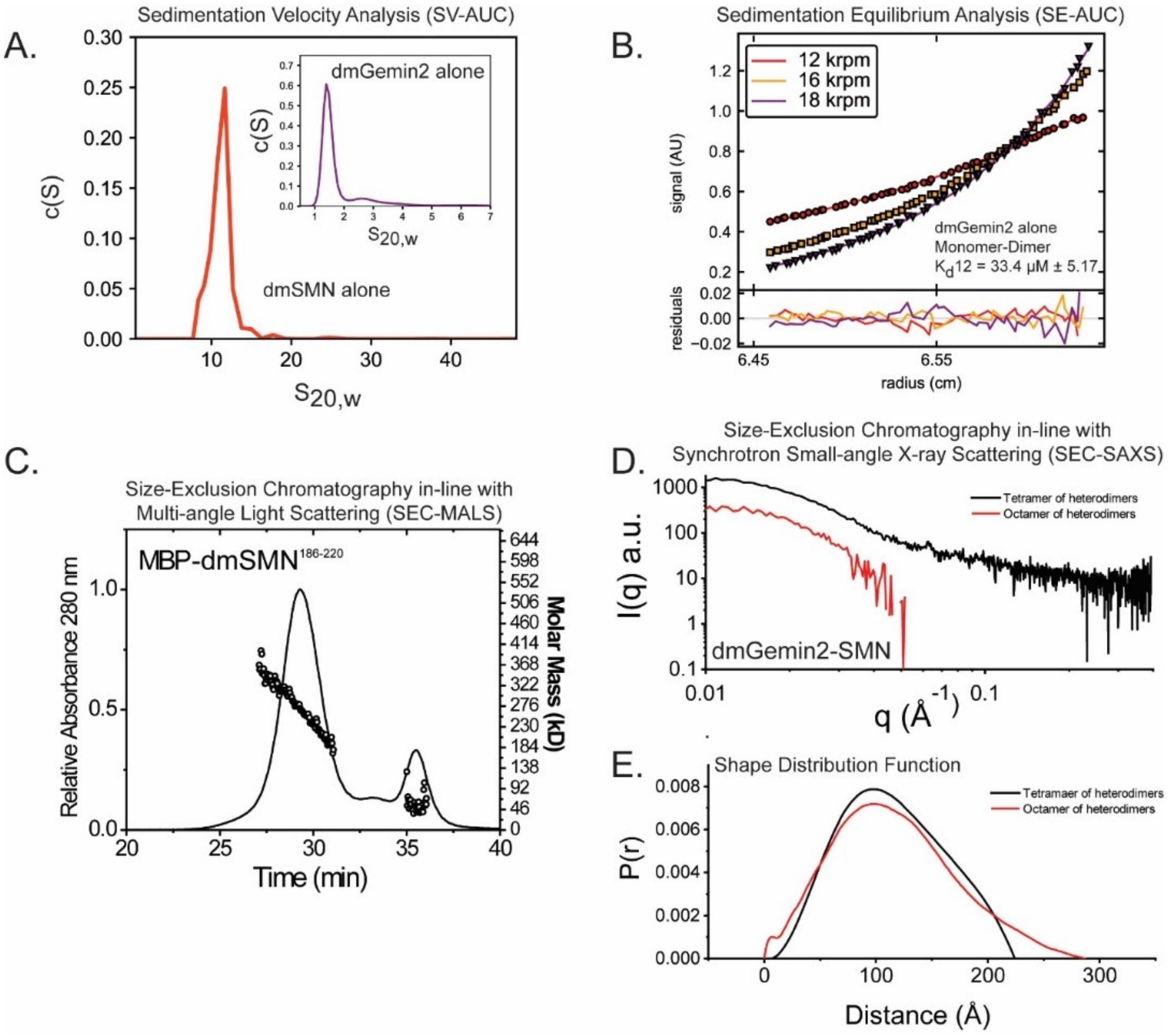

Figure S2. Biophysical characterizaton of *Drosophila* SMN and Gemin2. ***A***. Sedimentation velocity analysis of dmSMN and dmGemin2 at 20ºC. c(S) distributions derived from the fitting of the Lamm equation are shown for dmSMN (red) and dmGemin2 (blue, inset panel). This analysis of dmSMN shows evidence of a large ~11S species consistent with studies of the SMN•G2 complex from different species. The analysis of dmG2 alone shows evidence of a mostly monomeric species with some evidence of an oligomer. ***B***. Sedimentation equilibrium analysis of dmGemin2 at 4ºC. Shown in the upper panels are radial absorbance data plotted with fitted models shown as solid lines for each of three speeds. Shown in the respective lower panels are the residuals for each fit. Data were best described with a monomer-dimer fit with a determined K_d of 33.4 μM ± 5.2. C. SEC-MALS analysis of MBP-dmSMN$^{186-220}$. Shown as a black line is the absorbance profile of protein as a function of retention time in a Superdex-200 10/300 column at room temperature (left axis). Black circles denote molecular masses determined by in-line light scattering (right axis). The mass profiles determined span from dimer to octamer in the first peak and monomer in the second peak. D. SEC-SAXS analysis of dmSMN•G2. E. Resulting deconvoluted scattering profiles from SVD-EFA analysis of SEC-SAXS data collected for dmSMN•G2. Mass calculations using Q_r and Porod volume relationship confirm the presence of tetrameric and octameric species. Model-independent properties derived from this analysis are shown in Supplemental Table 3. F. Shape Distribution Function analysis of tetramers (black) and octamers (red) of dmSMN•G2. Relates to Fig. 2.

A

Vertebrate SMN orthologs:

```
Human        WLPPFPSGPPIIPPPPPICPDSLDDADALGSMLISWYMSGYHTGYYMGFRQNQKEGRC---SHSLN--
Bird         WPPPFPAGPPLIPPPPPMGPDSPEDDEALGSMLIAWYMSGYHTGYYLGLKQSRMEAAL---EREAYLK
Snake        WSPPFPSGPPLIPPPPLLSSDSPDDDEALGSMLIAWYMSGYHTGYYLGLKQGRMEATL---ERHAHSK
Turtle       CLPPFPTGPPLIPPPPPMGPDSPEDEEALGSMLIAWYMSGYHTGYYLGLKQSRMEAAL---ERCPHPK
Toad         SLPPPPP-FFSTEWEEYDEEVEEQDEDALACMLMAWYMTGYHTGFYMGLKQGRAEALRttcKKGSRRK
Frog         WPPPFLPGPPMIPPPPPMSPDACEDDEALGSMLIAWYMSGYHTGYYLGLKQGRMESSF---GKSPHQK
Coelacanth   VPSWPPVIPPFPPPPPPVTPEFDDADSTLVCMLLAWYMSGYHTGYYMGLKHGQAKATGssqKKHPKRK
Fish         WPPMIPLGPPMIPPPPPMSPDFGEDDEALGSMLISWYMSGYHTGYYMGLRQGRKEAAA---SKKSHRK
Shark        CLPTIPGGPPLIPPPPPM [6] EDDEALGSMLIAWYMSGYHTGYYLGLKQGRAEEALgksSHRGSLSPKERV
VertCons     WPPPFPxGPPLIPPPPP  [6] EDDEALGSMLIAWYMSGYHTGYYLGLKQGRxEAxx---xKxxxrK
D.melano     VMPPMPPVPPMIV      [6] EQD--FVAMLTAWYMSGYYTGLY----QGKKEASTtsgKKKTPKK
  vSmnEAL    VMPPMPPVPPMIV      [6] EQDEALVSMLTSWYMSGYYTGYYMGLRQGKKEASTtsgKKKTPKK
  Y motif                           LxxxLxxxYxxxYxxxYxxxL
  G motif                                   GxxxGxxxGxxxG
  s motif                             sxxxsxxxsxxxT
```

B

Fungal SMN orthologs:

```
S.pombe        EFMEVPPPI            [5]   DETYKKLIMSWYYAGYYTGLAEGLAKSEQRKD
Truffle        PPPGLPPIP            [8]   NEVLRNLMMSWYYAGYYTGLYEGQQQRQHGKN
Heterostel  LPPPPPFPPLPP            [4]   NDELGDLLLSWYYSGYYTGIYQERKRQRGEAAASNHHMNQ
Dictyostel  PPPTPTNYPP[6]PPPMP[6]         DDELADLLLSWYYSGYYTGVYQERKRNSRLNQTPHPNHIN[69]
Neurosp     PGPPLML                 [4]   DEELKKLLMSWYYAGYYTGLYEGKQKALHEQAQQ
B.bassian   PVISPQQALL              [4]   DDNLRKLLMSWYYAGYYTGLHEGQQQQTQAQQQPQ
Aspergil    PEVSVQGTNTTDGP         [16]   DEGLKNLMMSWYFAGYYTGLYEGQQRANQNKSS
Lichen      PGAPTMPNAVL             [4]   DEALKNLMMSWYFAGYYTGLYEGQQQAAQTPAQRPSGDRKEAD
P.italicum  PAMPMPHPIM              [4]   DESLKNLMMSWYYAGYYTGLHEGQQQTNSNQSS

FungCons    PxxPPPxxPx              [n]   DExLKNLLMSWYYAGYYTGLYEGQQRxxxxxxx
```

Figure S3. Alignment of YG box sequences from vertebrate and fungal SMN orthologs. ***A.*** Comparison of SMN YG boxes from nine different vertebrate clades showing the overall vertebrate consensus (VertCons). The three sequence motifs (Y, G and s) identified in Fig. 1A are shown below for comparison. Note the apparent conservation of a fourth GxxxG repeat (within context of QGRxE) among the vertebrates that provides motivation for insertion of MGLR sequence into *Drosophila* (D.melano) SMN to create vSmn and vSmn^{EAL} (relates to Fig. 5A). A conserved histidine (His273 in human) is shaded in gray. Two poorly conserved cysteine residues in human SMN are shown in red text, one of which (human C289A) was mutated in Fig 7D to create the ΔCys construct. ***B.*** Comparison of nine different fungal orthologs, revealing the overall lack of conservation of Ala141 (highlighted in yellow, relates to Fig. 6A). The fungal consensus sequence (FungCons) is presented below.

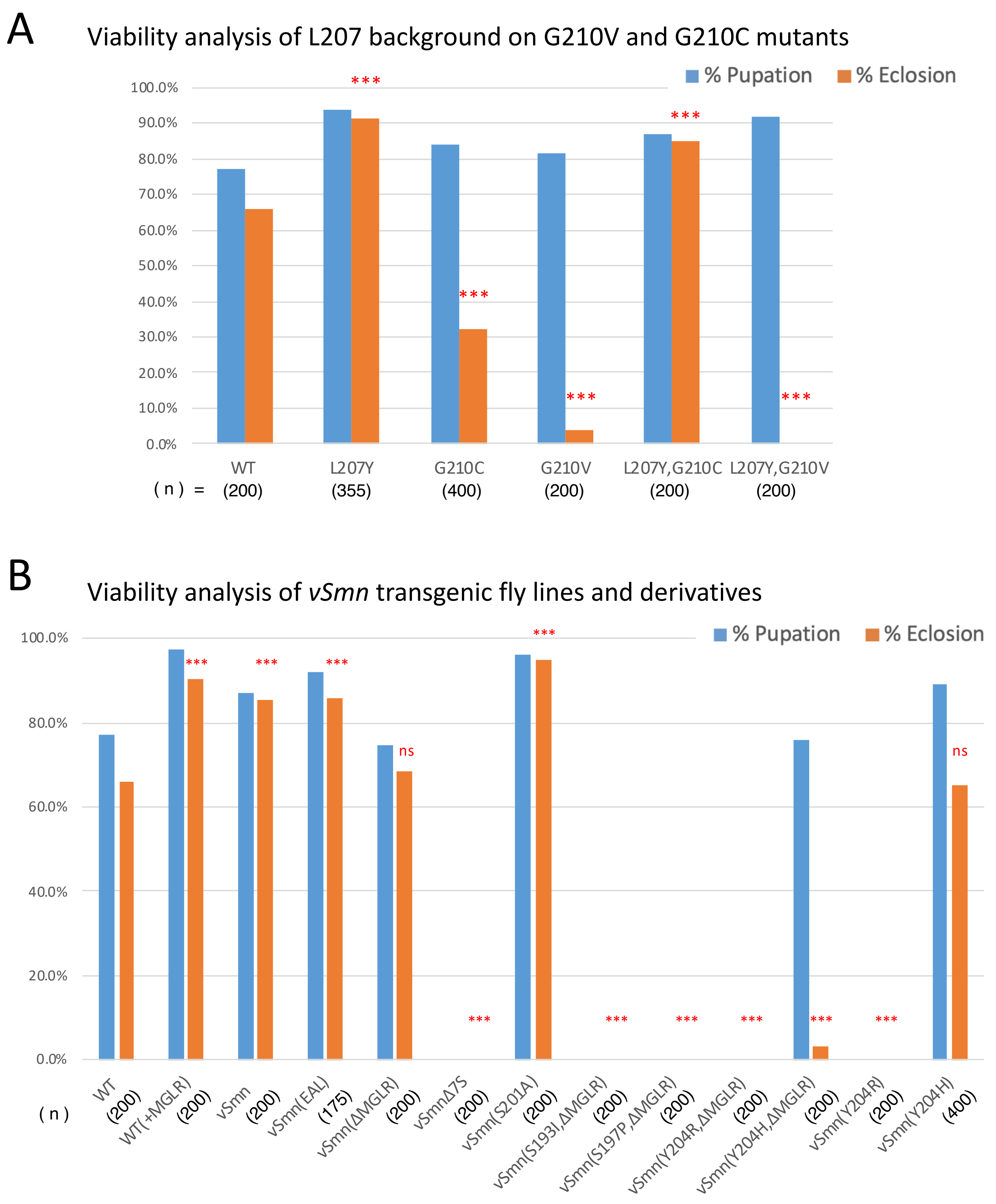

Figure S4. Pupal and adult viability analysis of Flag-Smn transgenic lines. ***A.*** Comparison of pupation and eclosion frequencies of wild-type (WT) and Smn missense mutations (relates to Fig. 5A). ***B***. Comparison of pupation and eclosion frequencies of vertebrate Flag-Smn (vSmn) fly lines as compared to WT controls (relates to Fig. 5A). For panels *A* and *B*, the total number of animals scored for each fly line is shown in parentheses below the genotype. *p < 0.05, **p < 0.01, ***p < 0.001. p > 0.05 is not significant (ns)

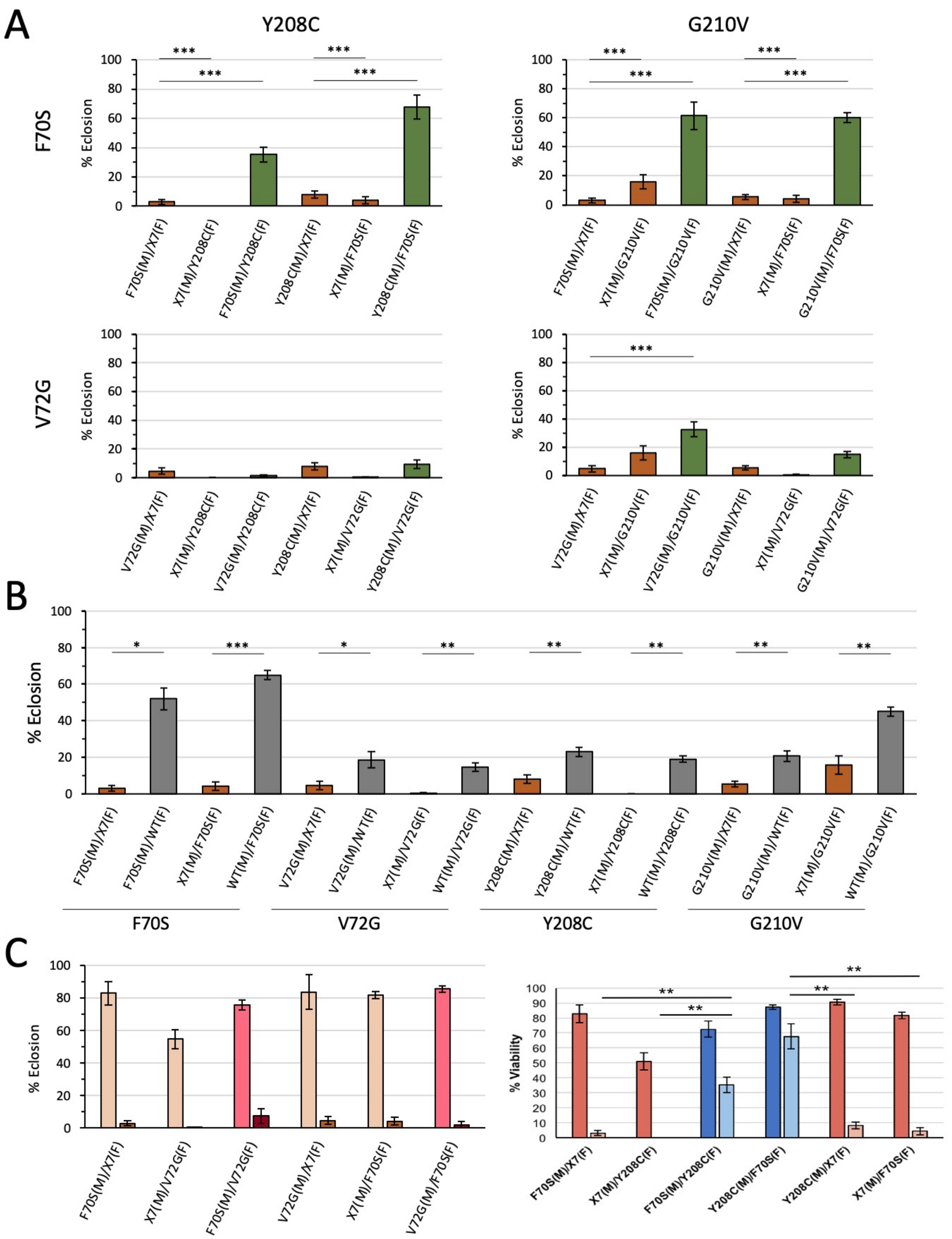

Figure S5. Intragenic complementation analysis. ***A-C.*** Adult eclosion frequencies of various combinations of SMA-causing point mutations in either the tudor domain (F70S and V72G) or the YG box (G210V and Y208C). The genotype of each parental strain (male, M or female F) is shown in parenthesis. For each cross n>200 animals. Experiments carried out as previously described (see Refs. 21, 44).

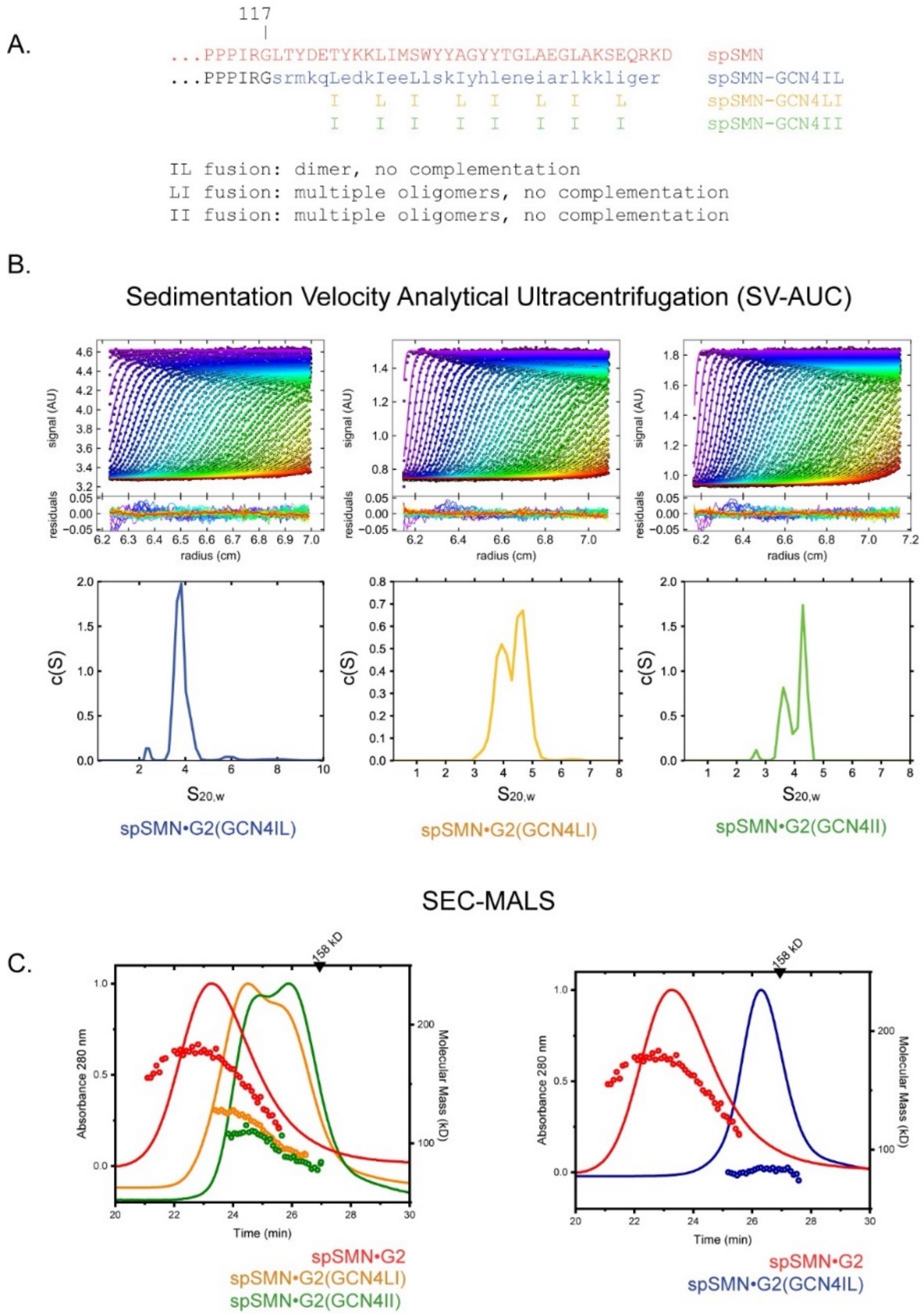

Figure S6. Analysis of spSMN(GCN4) chimeras. ***A***. GCN4 variants fused to spSMN•G2. Shown are the GCN4 variants substituted for YG box regions spSMN•G2 constructs, along with their respective behaviors in yeast complementation assays. ***B***. Sedimentation velocity analysis. For each construct, representative absorbance data (colored circles) for sedimentation boundaries are shown in the upper panels as a function of radial position and time. Shown in solid lines are the fits to the Lamm equation, as performed in SEDFIT (27). In the respective lower panels, the residuals of these fits are shown. c(S) distributions derived from the fitting of the Lamm equation are shown for each. This analysis shows evidence of mostly dimers of GCN4(IL) constructs, while GCN4(LI) (yellow) and GCN4(II) (green) constructs show behavior consistent with the occurrence of trimers and tetramers. ***C***. SEC-MALS analysis. Shown for each construct as a black line is the absorbance profile of protein as a function of retention time in a Superdex-200 10/300 column at room temperature (left axis), each injected at 8 mg/mL. Black circles denote molecular masses determined by in-line light scattering (right axis). Relative to wild-type complex (red), GCN4(IL) fusions (blue) are dimeric, while the mass profiles for GCN4(LI) (yellow) and GCN4(II) (green) constructs show evidence for higher order species approaching tetramer. All analyses here were performed in 20 mM Na/KPO_4 pH 7.0, 300 mM NaCl, and 2 mM DTT.

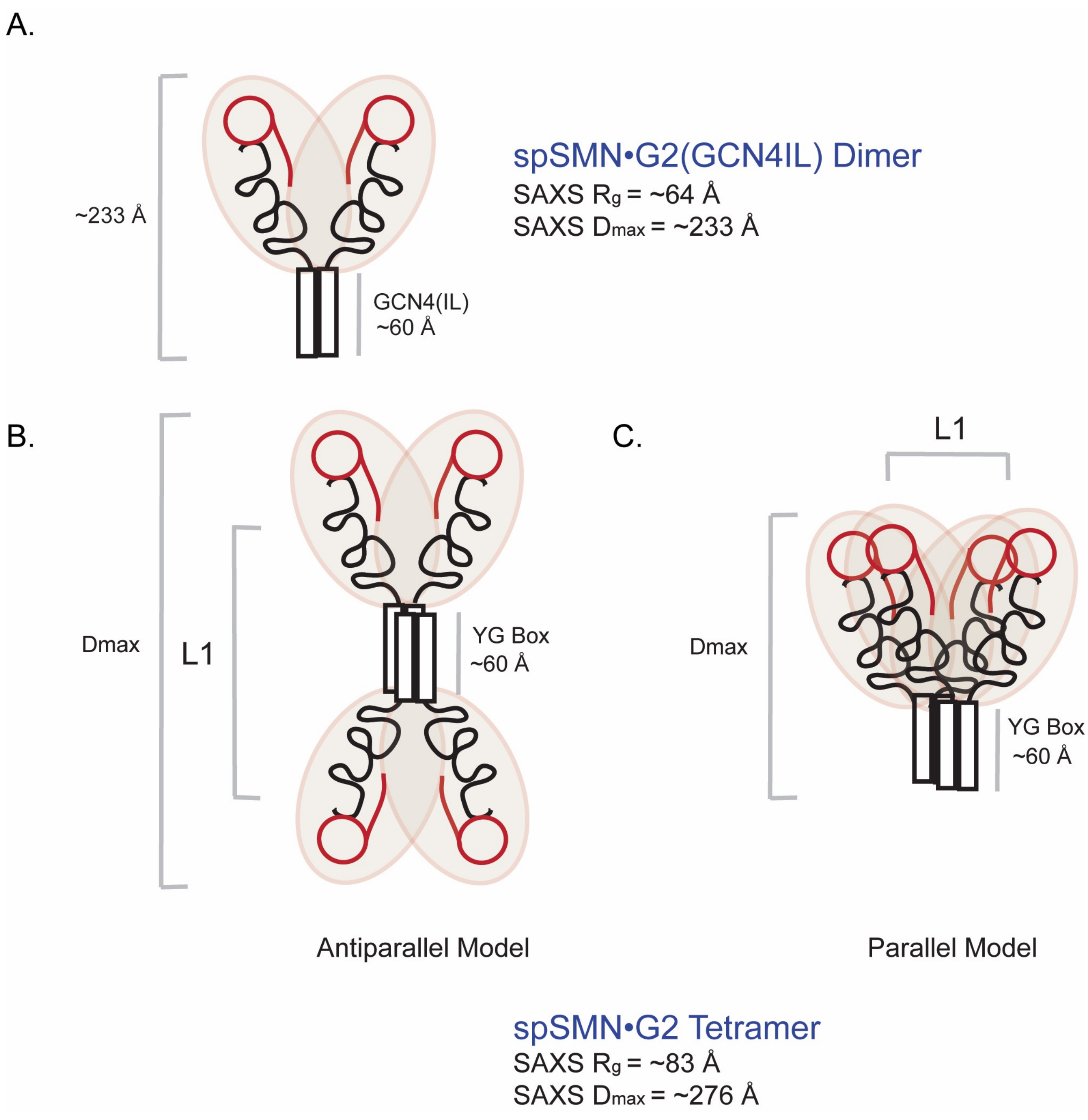

Figure S7. SAXS analysis of parallel and antiparallel tetramers. ***A***. The spSMN(GCN4)•G2 dimer properties were determined by SAXS (see Table S4). The length of the YG box is ~60 Å as observed by X-ray crystallography. ***B-C***. With the parameters for a spSMN(GCN4)•G2 model dimer, we can then consider two models for tetramerization: an antiparallel model (B) and a parallel model (C). The experimentally determined properties for the spSMN•G2 tetramer (Ref.) are: R_g =83 Å and D_{max}= 276 Å. The small increase in D_{max} for the tetramer vs. the dimer implies that the globular and unstructured regions of SMN•G2 are able to occupy a similar volume of space when two dimers associate to form a tetramer. This appears more likely in the parallel model, since the antiparallel model requires an additional 60 Å separation between the N-terminal domains. The experimental R_g values allow for calculation of L_1 (center of mass to center of mass distance) of ~106 Å using the Parallel Axis Theorem (see Methods). Either model would be consistent with this value; the 106 Å distance could be readily contained within a 233 Å N-terminal domain tetramer of the parallel model (C) or it could span the YG boxes to relate two N-terminal domain dimers (B). Relates to Fig. 7.

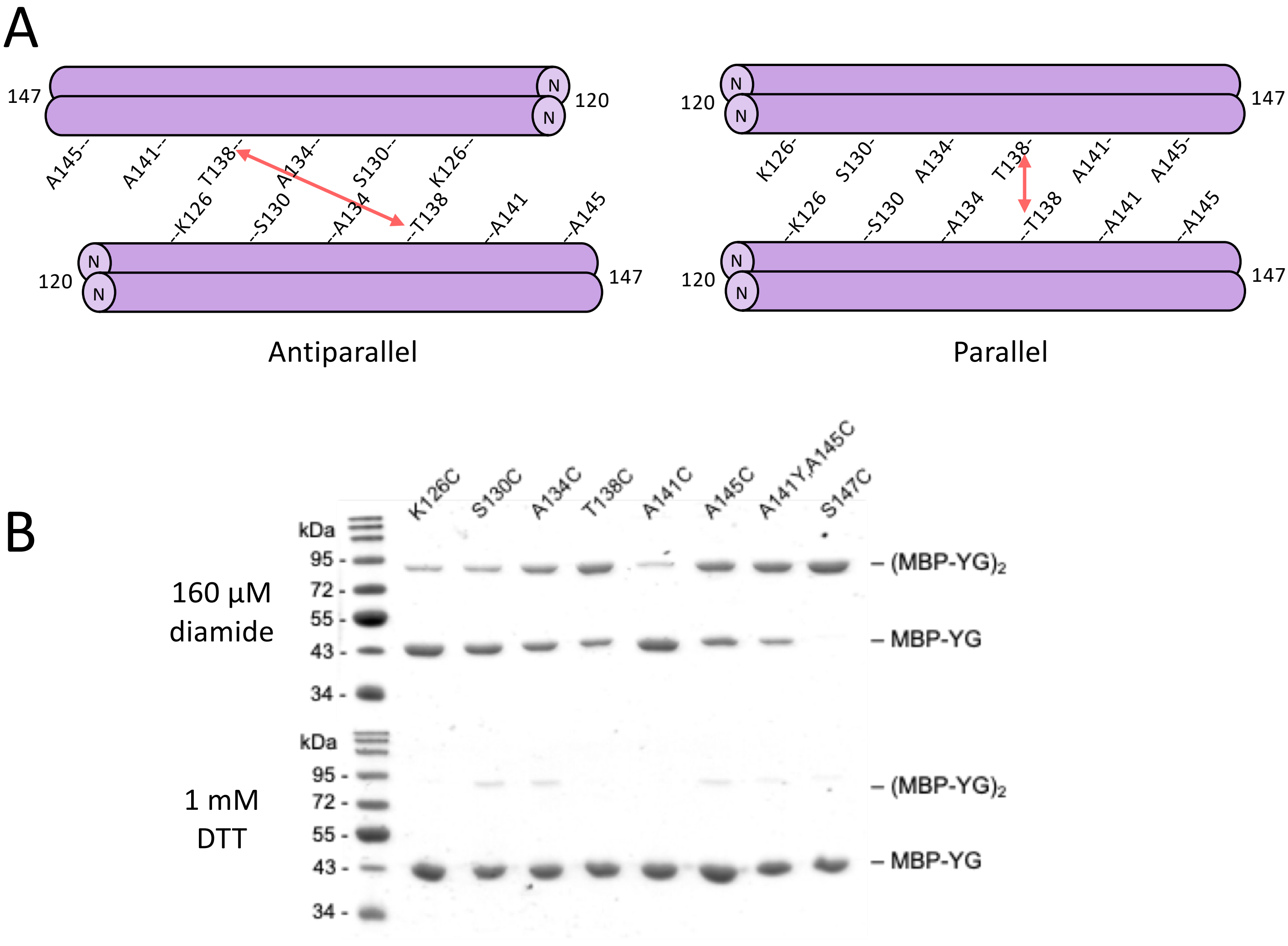

Figure S8: Disulfide crosslinking analysis of yeast MBP-YG box fusions. ***A.*** Cartoon of disulfide crosslinking between YG box dimers containing single cysteine substitutions at indicated residues. Crosslinking between Thr138 residues is illustrated with a red arrow as an example. Disulfide crosslinking allows for resolution of dimers upon non-reducing SDS-PAGE. The S147C substitution serves as a positive control because spS147 lies within the dimer interface and is efficiently crosslinked (Gupta et al., 2015). ***B.*** SDS-PAGE of MBP-spSMN YG box fusions treated with 160 µM diamide for 60 min (top) or 1 mM DTT (bottom).
